# Unveiling the Complexity of *KMT2A* Rearrangements in Acute Myeloid Leukemias with Optical Genome Mapping

**DOI:** 10.3390/cancers16244171

**Published:** 2024-12-14

**Authors:** Sandrine A. Lacoste, Vanessa Gagnon, François Béliveau, Sylvie Lavallée, Vanessa Collin, Josée Hébert

**Affiliations:** 1 Quebec Leukemia Cell Bank, Maisonneuve-Rosemont Hospital, Montréal, QC H1T 2M4, Canada; sandrine.lacoste.cemtl@ssss.gouv.qc.ca (S.A.L.); vanessa.gagnon2@hotmail.fr (V.G.); francois.beliveau.cemtl@ssss.gouv.qc.ca (F.B.); slavallee.hmr@ssss.gouv.qc.ca (S.L.); vanessa.collin.cemtl@ssss.gouv.qc.ca (V.C.); 2 Cytogenetics Laboratory, Maisonneuve-Rosemont Hospital, Montréal, QC H1T 2M4, Canada; 3 Division of Hematology-Oncology and Cellular Therapy, Maisonneuve-Rosemont Hospital, Montréal, QC H1T 2M4, Canada; 4 Department of Medicine, Faculty of Medicine, Université de Montréal, Montréal, QC H3T 1J4, Canada

**Keywords:** acute myeloid leukemia, *KMT2A* rearrangements, gene fusions, partial tandem duplications, optical genome mapping

## Abstract

**Background:** *KMT2A* rearrangements are major genetic entities in the classification of acute myeloid leukemias (AMLs), but their diverse and frequently cryptic nature makes their detection and characterization challenging. Karyotypic anomalies at the *KMT2A* locus and/or abnormal *KMT2A* Fluorescence in situ hybridization (FISH) results strongly indicate a *KMT2A* fusion, but the identification of the translocation partner gene often requires further investigation. *KMT2A* partial tandem duplications (PTDs), on the other hand, are undetectable by standard cytogenetics methods. **Methods:** We herein report the optical genome mapping (OGM) analysis of 38 AML samples: 12 cryptic/hard-to-characterize *KMT2A* fusions, 20 *KMT2A*-PTDs and 6 cases with no *KMT2A* anomaly. **Results:** In all the fusion cases, the rearrangement between 5’*KMT2A* and the 3’partner gene was identified as a translocation t(v;11q23.3)(v;118479068), and the analysis of co-occurring variants elucidated the formation of the rearrangement. The *KMT2A* variants detected in the *KMT2A*-PTD cases were surprisingly diverse. Combined with RNAseq data, OGM analysis identified 9 distinct in-frame *KMT2A*-PTD variants among the 20 cases analyzed. **Conclusions:** With the clinical development of menin inhibitors for the treatment of patients with *KMT2A*-rearranged acute leukemias, the characterization of these rearrangements is of utmost importance. Our results suggest that OGM is a promising tool for accurate genetic diagnosis in this context.

## Simple Summary

The classification of acute myeloid leukemias used to diagnose and inform patient care is primarily based on the identification of specific genetic changes in patient leukemic cells. The presence of a rearrangement in the *KMT2A* gene is frequently associated with a poor clinical outcome, but promising treatments targeting this type of abnormality are currently being evaluated and could greatly affect patients’ outcomes. It is therefore of the utmost importance to properly identify the presence of these rearrangements in patients. This is, however, a challenging diagnosis for clinical cytogenetics laboratories because such rearrangements are very heterogeneous in nature and can remain undetectable using conventional methods of detection. The aim of this study was to use optical genome mapping, a novel cytogenomic method, on samples with known genetic abnormalities to assess the ability of this technique to detect and fully characterize *KMT2A* rearrangements, which should ultimately help improve patient treatment.

## 1. Introduction

Rearrangements involving the *KMT2A*/*MLL* gene are recurrently occurring in adult acute myeloid leukemia (AML) and constitute a defining genetic abnormality in the fifth edition of the WHO classification [1]. The identification of the specific *KMT2A* rearrangement (*KMT2A*-r) influences risk stratification and has a high clinical significance for disease monitoring and therapy. Currently, the European LeukemiaNet (ELN) recommendations for AML classify translocations involving *KMT2A* as either intermediate-(t(9;11)(p21.3;q23.3)/*KMT2A*::*MLLT3*) or adverse-(t(v;11q23.3)/*KMT2A*-rearranged) genetic risk [2]. In-frame partial tandem repeats (called partial tandem duplications, PTDs) spanning variable N-terminal exons of *KMT2A* are another frequent form of *KMT2A*-r. *KMT2A*-PTDs are included in the IPSS-M prognostic model for myelodysplastic syndromes (MDSs) [3], but are not yet included in current AML classifications. However, *KMT2A*-PTDs are generally associated with a poor prognosis [4,5], and in some studies, with a benefit from hematopoietic stem cell transplantation in first remission [6], reinforcing the need for their identification for the proper clinical management of AML patients. This is especially true with the emergence of promising new treatments such as menin inhibitors that target *KMT2A*-r [7,8,9].

The detection and characterization of *KMT2A*-r remains a challenge for diagnostic laboratories. No less than 112 translocation partner genes have been identified to date for *KMT2A* [10]. Fusions can usually be detected as karyotypic aberrations involving the 11q23.3 chromosome band and/or rearrangements at the *KMT2A* locus identified with a Fluorescence in situ hybridization (FISH) break-apart probe. However, the *KMT2A* fusion partner frequently remains undetermined or uncertain, unless a complementary type-specific assay has been developed and validated to identify this partner gene [11]. The identification of new or rare partners can be achieved using long-distance inverse-PCR analysis [12], targeted DNA next-generation sequencing (NGS) of the *KMT2A* gene [13] or targeted RNA sequencing [14], but these options are not always accessible in a time frame that can inform patient care, and the cost–result ratio always needs to be considered (see a review of existing methods of detection in [15]). Some fusions, such as t(10;11)(p12;q23.3)/*KMT2A*::*MLLT10* (~13% of fusion cases in adult AML [16]), are especially challenging, as they tend to be cryptic by conventional cytogenetics methods and can remain entirely undetected [17]. Similarly, *KMT2A*-PTDs are not identified by karyotype or FISH and require specific assays such as targeted DNA NGS [16,18,19] or multiplex-ligation probe amplification (MLPA) for detection [20,21]. Although these additional molecular methods have been successfully used to date to identify the presence of *KMT2A*-r, they are not widely available in clinical laboratories.

In that context, optical genome mapping (OGM) can be a valuable tool in the characterization of *KMT2A*-r. This technique maps specific labeled sites along large molecules (>150 kb) of native genomic DNA. Combined with several algorithms for analysis and comparison to a reference genome, OGM can identify copy number variants (CNVs) as well as structural variants (SVs), such as insertions, deletions, inversions, duplications and translocations, in a single experiment and in an unbiased manner. OGM is currently being evaluated for the clinical management of hematological malignancies and has demonstrated its ability to detect *KMT2A* fusion genes and PTDs [21,22,23,24,25,26]. In addition to providing clinically relevant information without the need to select specific targets to analyze, OGM data constitute a unique opportunity to bridge the gap between standard cytogenetics and molecular data and to understand the events associated with the formation of *KMT2A*-r. In this work, we present the OGM analysis of 38 AML samples: 12 cryptic or hard-to-characterize *KMT2A* fusions, 20 *KMT2A*-PTD cases as well as 6 AMLs with no evidence of any *KMT2A* anomaly.

## 2. Materials and Methods

### 2.1. Sample Selection

Bone marrow (BM) or peripheral blood (PB) samples from AML patients were collected between 2002 and 2022 at the Quebec Leukemia Cell Bank, which is certified by the Canadian Tissue Repository Network. Specific samples were selected for OGM analysis based on available information regarding the presence or not of *KMT2A* rearrangements (Table 1 and Table S1). Karyotype information, *KMT2A* FISH results (Vysis LSI MLL Dual Color, Abbott Molecular, 05J90-001, Des Plaines, IL, USA) as well as fusion transcript data (FusionCatcher v.1.10 [27] analysis on RNAseq data) were used to select 12 cases presenting cryptic or hard-to-characterize *KMT2A* fusions. Some of these cases have been described previously [28]. Cases presenting a *KMT2A*-PTD (*n* = 20) or not (*n* = 6) were selected based on the presence of aberrant exon-exon junctions in the N-terminal portion of *KMT2A* identified in unaligned RNAseq reads [28,29]. The results of transcript analyses for the selected samples are presented in Table S2. *FLT3*-ITD and *NPM1* mutation status, as determined with laboratory-developed clinical tests (target-specific PCR amplification and fragment analysis or NGS), was also available for most samples (Table S1).

### 2.2. OGM Sample Preparation and Data Acquisition

Supplies and procedures for optical genome mapping were used and applied as recommended by the manufacturer (Bionano Genomics, San Diego, CA, USA). Briefly, cryopreserved samples of mononuclear cells of BM or PB were thawed at 37 °C and washed in cold RPMI. Ultra-high molecular weight genomic DNA of 1.5 million viable cells was then isolated using the SP Blood and Cell Culture DNA Isolation kit (G1 #80030 or G2 #80060), then fluorescently labeled at DLE-1 sites (CTTAAG) and stained with the DLS labeling kit (G1 #80005 or G2 #80046). Data acquisition (~1500 Gb/sample) was performed with G2.3 (#20366) or G3.3 (#20440) Chips on the Saphyr^®^ System (Bionano Genomics, San Diego, CA, USA).

### 2.3. OGM Data Analysis

The Rare Variant Analysis (RVA) pipeline was performed using Solve v3.8.1 with hg38_DLE1_0kb_0labels_masked_YPARs.cmap as a reference. Data were imported and processed through the VIA Software v7.0 [30] using the “AML hg38” sample type with default settings (Table S3). Quality control values for sample analysis are presented in Table S4. Default filters were used to identify SVs with recommended VCF filter values and exclude germinal variants listed in the OGM database of 394 phenotypically healthy individuals. For each sample, the AML-specific filters that are integrated by default in the “AML hg38” sample type in VIA (to prioritize the analysis of known AML-related genes/regions) were unselected in order to review all identified SVs or CNVs individually. Variant validation consisted of (1) excluding likely artifacts (variants overlapping CNV- or SV-masked regions) and (2) identifying likely benign variants (variants in regions with no gene/in intronic gene regions/affecting genes unlikely to be relevant to malignant transformation based on the CIViC knowledgebase [31] included in the “AML hg38” sample analysis). Considering their potential contribution to malignant transformation, large copy number variants (>500 kb) and translocations were not tagged as likely benign, even if no relevant genes were identified [25]. Allelic variants (allelic imbalance (AI) or absence-of-heterozygosity (AOH)) with a size ˂ 15 Mb were excluded as likely false positives, unless they were validated by the presence of a corresponding copy variant. All validated variants (including likely benign) are listed in Table S5. When indicated, the acquired data were reanalyzed with the Guided Assembly—Low Allele Fraction pipeline to better characterize some variants of interest. This new analysis pipeline available with Solve v3.8.1 is a type of de novo assembly adapted for the analysis of cancer genomes with improved performance for the detection of some SVs, including insertions and duplications. Variants of interest identified in VIA were also visualized in Bionano Access 1.8.1.

The genomic coordinates are according to the GRCh38 reference genome and Matched Annotation from NCBI and EMBL-EBI (MANE) transcripts [32] were used as a reference. For *KMT2A*, the reference transcript was NM_001197104 and its UniProt protein match Q03164-3.

### 2.4. Sanger Sequencing Validation of Newly Identified Fusion Genes

For *KMT2A* fusion rearrangements with no RNAseq data available, the expression of the identified or suspected fusion gene determined by OGM was validated by RT-PCR (OneTaq One-Step RT-PCR Kit, New England Biolabs, Whitby, ON, Canada) followed by Sanger sequencing of the amplicon. The primers for *KMT2A*::*MYCBP* (case 21H024) were the following: 5′-TGTTTCCTGATGACATGCCCA-3′ (forward) and 5′-CCTATTCAGCACGCTTCTCCT-3′ (reverse). The primers for *KMT2A*::*CBL* (case 07H114) were the following: 5′-CTTGCTCCACCCATCAAACC-3′ (forward) and 5′-TTTTCTGACTCCTCGGGACC-3′ (reverse).

## 3. Results

The identification of *KMT2A*-r is important for AML diagnosis and to determine eligibility to targeted treatments. In this work, we focused on a series of cases with known status for *KMT2A*-r but that are not fully characterized and used optical genome mapping to identify the genetic events involved in the rearrangements. The characteristics of the selected samples are presented in Table 1 and Table S1.

### 3.1. Position of Labeled Sites in the KTM2A Gene

**Figure 1:**
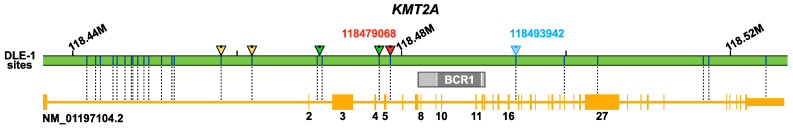
Map of fluorescently labeled DLE-1 sites in *KMT2A*. Colored triangles indicate DLE-1 sites of interest in the characterization of *KMT2A* variants. The major breakpoint cluster region (BCR1) in the *KMT2A* gene (intron 7 to exon 13) is indicated in gray with a darker shade for the area with the highest density of identified breakpoints (intron 9 to intron 11) [16]. The red- and blue-colored numbers above the triangles are the GRCh38 genomic coordinates for the DLE-1 sites on either side of the BCR1.

OGM is based on the imaging and analysis of fluorescently labeled sites (14–15 labels per 100 kb on average) in native genomic DNA. Variants are identified by a comparison of the pattern of these labeled sites in patient DNA (patient maps), with the expected pattern of those sites predicted from the reference genome. As a result, the frequency and location of labeled sites in any specific region can affect the performance of the technique. The map of labeled sites in *KMT2A* is presented in Figure 1 and the sites most important to understand *KMT2A*-r are indicated with colored triangles to facilitate their identification in patient maps (Figure 1).

The major breakpoint cluster region (BCR1), where the vast majority of *KMT2A* fusions occur in adult AML [16], is entirely contained in a 14.9 kb region between two DLE-1 sites (red and blue triangles in Figure 1). As a result, any translocation occurring in the BCR1 is likely to be detected by OGM as a rearrangement within the region delimited by these two sites, but without more information regarding the specific breakpoint location. Similarly, there are very few sites in the region most frequently rearranged in *KMT2A*-PTDs (exons 2 to 10). This is expected to have consequences both for the identification of any repeat pattern by the OGM algorithms and for the determination of what specific exons are duplicated. Despite these limitations, the OGM data obtained on very long molecules of native DNA generate important information on the genomic context of specific rearrangements.

### 3.2. KMT2A Fusions

Twelve cases with *KMT2A* fusions were selected for OGM analysis, seven of which presented no karyotypic anomaly at the *KMT2A* locus (11q23.3 cytoband). The presence of the rearrangement in these cases was determined by an abnormal *KMT2A* break-apart FISH result, with the exception of two samples that also presented normal FISH results (Table S1) but have been previously identified as *KMT2A* fusions by transcriptomic analysis [28]. Evidence of a *KMT2A* fusion transcript was also available for 9 of the 12 selected cases (Table S2).

The variants identified by OGM and processed as the “AML hg38” sample type in VIA are displayed by default in the software with the list of overlapping CIViC genes, allowing an easy identification of all variants in the vicinity of the *KMT2A* locus on chromosome band 11q23.3 (see variants list exported from VIA in Table S5). At least one translocation (interchromosomal) or fusion (intrachromosomal) variant indicating the presence of a *KMT2A*-r was identified in each case analyzed. The genome mapping nomenclature guidelines recommend to indicate the nucleotide coordinates at the chromosome breakage sites for the translocations detected by OGM [33]. This information is provided as “ISCN Nomenclature” by the analysis software (Table S5) and corresponds to the DLE-1 site identified in the patient map that is the closest to the breakpoint of the translocation. As expected from the map of DLE-1 sites in *KMT2A*, all the OGM variants depicting a rearrangement of the 5′ region of *KMT2A* with a 3′ fusion partner in our cohort had the format t(v;11q23.3)(v;118479068), with the nucleotide coordinate in chromosome 11 being the coordinate of the DLE-1 site immediately upstream of the BCR1 (indicated by a red triangle in Figure 1). In all 9/12 cases where the information was available, the rearrangement identified by OGM was concordant with the gene fusion expected from the transcript analysis.

The genetic events occurring at the *KMT2A* and the 3′ partner gene loci, as well as other genetic events associated with the *KMT2A*-r for the 12 cases analyzed (in particular those explaining atypical FISH results), are summarized in Table 2. Many of the fusions analyzed in this work (7/12) are fusions with *MLLT10* which, although relatively frequent in leukemia (~13% of *KMT2A* fusions in adult AML as reported by Meyer et al. [16]), tend to be cryptic. Indeed, 26% of *KMT2A*::*MLLT10* identified over a 10-year period by Petterson et al. [17] presented no structural or numeric anomaly involving chromosome 10 (*MLLT10* locus at 10p12.31) or 11 (*KMT2A* locus at 11q23.3).

The events identified in the 12 cryptic or hard-to-characterize selected cases illustrate a variety of strategies in the formation of *KMT2A* fusions and are presented in detail in Figures S1–S12 and Figure 2.

The two *KMT2A* fusion cases that were only identified by their transcriptomic signature (no karyotypic nor FISH anomaly) were confirmed to have *KMT2A*::*MLLT10* fusions by OGM. Analysis of sample 09H102 indicated a translocation of 3′*MLLT10* into the *KMT2A* BCR1 with no evidence of copy change in either of these loci (Figure S1), whereas case 11H095 presented a tandem duplication of 5’*KMT2A* associated with the insertion of a copy gain of 3′*MLLT10* (Figure S2). In both cases, the consequences of the rearrangement on the DNA molecule were not sufficient to affect the *KMT2A* break-apart FISH results as the 5′ and 3′ signals remained in close vicinity on the *KMT2A* locus.

Another type of rearrangement identified involved the occurrence of a reciprocal t(10;11)(p12.31;q23.3)/*KMT2A*::*MLLT10* translocation that was associated with a complementary reciprocal t(5;10)(q15;p14) translocation (case 22H021, Figure S3). Taken together, these results explained the identification of a t(5;11) translocation by conventional cytogenetic analysis (Figure S3e). In case 21H024, an additional 3′ FISH signal was found to be caused by a distal copy gain of 11q that included part of the 3′ FISH probe target sequences downstream from the *KMT2A* locus (Figure S4). Importantly in this case, we identified a t(1;11)(p34.3;q23.3) *KMT2A*::*MYCBP* fusion confirmed by RT-PCR and Sanger sequencing. The resulting KMT2A-MYCBP protein fusion adds a dimerization domain to the N-terminal part of KMT2A (Figure S4f), which is a frequent feature observed for many uncommon KMT2A fusion proteins [34]. To our knowledge, this fusion has never been reported previously.

The analysis of cases with an additional 5′ FISH probe signal revealed the translocation of a copy gain of 5′*KMT2A* into the 3′ gene partner locus (*ENAH* in case 02H033 or *MLLT10* in case 06H073, Figures S5 and S6, respectively). Alternatively, an extra signal in case 18H072 (Figure S7) was possibly generated by a second rearrangement affecting the sequences targeted by the 5′ FISH probe upstream of the *KMT2A* locus (Figure S7d). However, the *KMT2A*::*SEPTIN6* fusion gene itself was associated with the fusion signal observed on the der(X), where the 5′*KMT2A* region is inverted in relation to the 3′*KMT2A* region (Figure S7d). The observed location of a 5′ signal on der(12) on metaphase FISH (Figure S7f) could not be entirely explained at the genomic level, but is likely related to the three-way translocation t(X;11;12) that remained undetected by OGM.

As could be expected, the loss of a 3′ FISH signal was associated with the deletion of the 3′*KMT2A* region in cases 07H160 and 10H031 (Figures S8 and S9, respectively). Interestingly, in case 10H031, this loss was accompanied by a copy gain of the 3′ partner gene *AFDN* and led to a cryptic t(6;11)(q27;q23.3)/*KMT2A*::*AFDN* fusion with a normal karyotype.

The next cases analyzed showed complex rearrangements which have been elucidated at least partially with OGM data. In case 05H128 (Figure S10), 5′*KMT2A* was deleted from chromosome 11 and transferred to the region of deleted 5′*MLLT10*, explaining the 5’ FISH signal detected on der(10). Case 06H077 revealed multiple genetic events resulting in the fusion gene, including the following: (1) the deletion of the *KMT2A* locus from chr11 (loss of 3′ FISH probe signal), with part of the 5′*KMT2A* probe sequences upstream from *KMT2A* remaining in place (5′ FISH probe signal on der(11)); (2) the insertion of a small 107 kb 5′*KMT2A* region from the deleted *KMT2A* locus into the *MLLT10* locus (5′ FISH signal on der(10)); (3) a 9.1 Mb inversion in chromosome 10 with a breakpoint into *MLLT10,* where the 107 kb region of 5’*KMT2A* was inserted next to 3′*MLLT10* (Figure S11).

**Figure 2:**
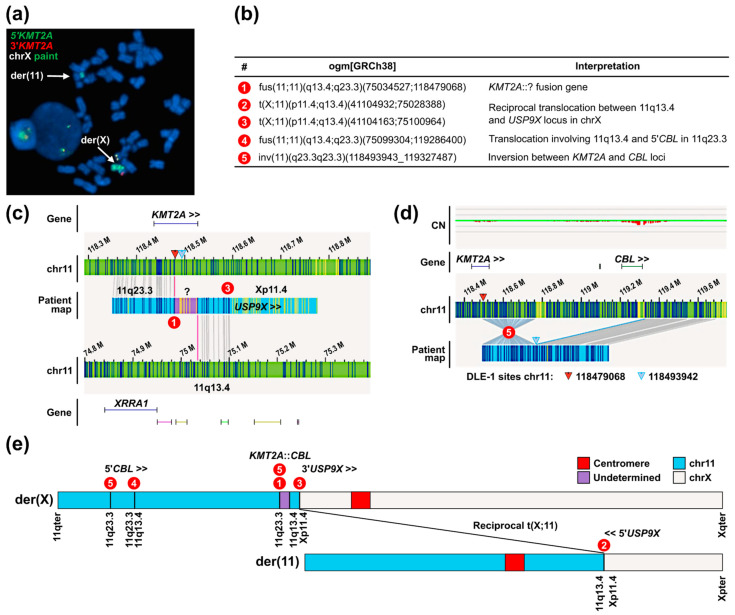
*KMT2A*::*CBL* rearrangement and putative derivative chromosomes in case 07H114. (**a**) *KMT2A* break-apart and whole chromosome X painting FISH probes on metaphase showing 1 fusion signal with 5’ and 3’ signals located on der(X). (**b**) Validated variants associated with the *KMT2A*-r and their interpretation. (**c**) *KMT2A* fusion gene (DLE-1 site 118479068 in red) in Access identified as a fus(11;11) variant with an unidentified region (in purple) between the 2 regions involved in the translocation. (**d**) Inversion between the *KMT2A* and *CBL* loci in Access. The presence of *CBL* as the fusion partner (located in unidentified purple region in panel c) was confirmed by RT-PCR and Sanger sequencing (Figure S12). (**e**) Putative der(X) and der(11) consistent with all data available for this case. Numbers in (**c**–**e**) correspond to variant calls listed in panel (**b**).

The last fusion case (case 07H114) presented in Figure 2 and Figure S12 illustrates the usefulness of OGM when trying to reconstitute how a specific rearrangement occurred using information from other identified variants in the sample. The FISH results for this case indicated the presence of a *KMT2A* rearrangement, with the 5′ and 3′*KMT2A* FISH signals both located on derivative chromosome X (Figure 2a). No 3′ fusion partner could be identified by OGM and no transcript data were available for this case. As in all other fusion cases, a translocation variant involving the DLE-1 site upstream from the BCR1 (Figure 2b–d, red triangle) was identified, suggesting the presence of a fusion gene. In this sample, the corresponding OGM variant call was a fusion (an intra-chromosomal translocation) between 5′*KMT2A* and another part of chromosome 11 presenting no known gene (Figure 2c). The co-occurrence of an inversion between the *KMT2A* and *CBL* loci (Figure 2d) led to the hypothesis that *CBL* could be the fusion partner located in the unidentified region of the fus(11;11). This hypothesis was confirmed by RT-PCR and Sanger sequencing (Figure S12d). A map of the putative derivative X and 11 chromosomes consistent with the available data is proposed in Figure 2e.

### 3.3. KMT2A-PTDs

Twenty cases with a *KMT2A*-PTD rearrangement and six without this genetic anomaly were selected based on the detection or not of aberrant exon-exon junctions involving the N-terminal part of *KMT2A* [28,29]. In all positive cases, the identity of the exons involved in the *KMT2A*-PTD was inferred from the nature of the junctions detected (Table S2). Based on this information, our OGM analysis was performed on *KMT2A*-PTD cases with the involvement of exons 2 to 8 (*n* = 9), exons 2 to 10 (*n* = 8) and other less common types of rearrangements (*n* = 3). Among the latter cases was a sample that presented several junction types, suggesting that more than one variant was present (case 18H194).

The circos plots for all the analyzed samples are presented in Figure S13 (controls) and Figure S14 (*KMT2A*-PTDs). As expected, the six control samples did not present any variant at the *KMT2A* locus. Importantly, *FLT3*-ITD mutations, which are frequent in both the control and *KMT2A*-PTD cases (Table 1), were not detected by OGM analysis because their size was below the detection limit of this technique. Overall, the OGM analysis identified a relatively small number of variants (Figure S14), although some events of interest were observed, such as *RUNX1* rearrangements in three of the *KMT2A*-PTD cases. The exact breakpoints in the *RUNX1* gene could not be determined precisely with the OGM data, but the variants identified in these three cases all affected at least one exon of the gene and have the potential to be pathogenic (Figure S15). Interestingly, *RUNX1* mutations have also been reported in ~25% of AML with *KMT2A*-PTD [4].

**Figure 3:**
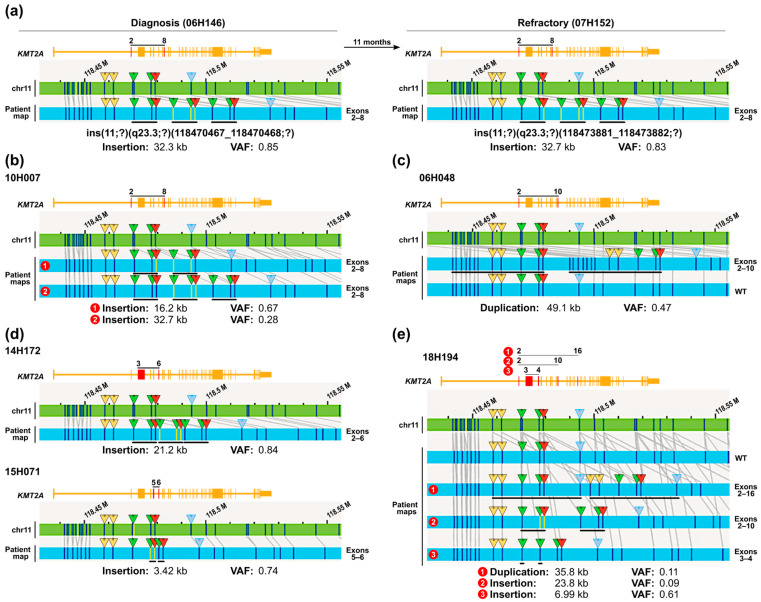
Repeat patterns of DLE-1 sites observed for *KMT2A*-PTD variants analyzed by OGM. (**a**) The same variant identified at 2 different time points for the same patient consisting of 3 copies of a pattern of DLE-1 sites consistent with the presence of exons 2 to 8 in each copy. (**b**) A similar pattern (involving exons 2 to 8) identified both as 2 copy and 3 copy formats in sample 10H007. (**c**) *KMT2A*-PTD involving exons 2 to 10 identified only with Guided Assembly—Low Allele Frequency analysis (wild-type allele also displayed). (**d**) Detection of less common variants involving repeats of exons 3 to 6 and exons 5 to 6. (**e**) Confirmation by OGM analysis of the simultaneous presence in the same sample of several unrelated variants that have also been detected as aberrant exon-exon junctions in RNAseq reads (result of Guided Assembly—Low Allele Frequency analysis is displayed). Variants were detected as insertions and/or tandem duplications. Black lines below patient maps indicate the DLE-1 sites included in the repeats. Colored triangles indicate DLE-1 sites of interest in the characterization of *KMT2A* variants.

Two of the selected *KMT2A*-PTD samples (involving exons 2 to 8) were collected from the same patient at time of diagnosis (06H146) and 11 months later at the refractory stage (07H152). An identical *KMT2A* variant was found in both samples, although the predicted position of the insertion site from the OGM analysis was slightly different (Figure 3a). The pattern of the DLE-1 sites in these variants was consistent with three tandem repeats of a region containing exons 2 to 8. The difference in the estimated coordinates of the insertion site is likely related to which DLE-1 sites in the patient map were formally identified or not by the algorithm, but the analysis clearly identified the same DNA molecule. Overall, the variants identified at both time points were the same, with the exception of two minor variants present at diagnosis that could not be detected later at the refractory stage (Table S6).

A similar pattern of three repeats was observed in all other *KMT2A*-PTD variants involving exons 2 to 8 (Figure S16a), with the exception of one sample with two repeats (08H063) and one with four repeats (05H111) of a specific pattern of DLE-1 sites. There was also a sample with both two and three copies of the same repeated region (10H007) (Figure 3b). An absence-of-heterozygosity (AOH) along the whole 11q arm was also detected in this last sample, suggesting that the two variants identified in this patient might have the same origin.

*KMT2A*-PTDs involving exons 2 to 10 had a more classical pattern of tandem duplications (*n* = 7), except for one sample with three repeats (06H090) (Figure S16b), but the DLE-1 sites involved in the repeat patterns were overall the same as for the 2 to 8 exon variants described previously (Figure S16a). Importantly, the *KMT2A*-PTD variant was not detected by the Rare Variant Analysis (RVA) pipeline in one of our samples (06H048), but was identified when the data were reanalyzed with the Guided Assembly—Low Allele Frequency pipeline of analysis, which is a new type of de novo assembly adapted to cancer genomes (Table S7, Figure 3c). It is unclear why the RVA pipeline did not detect this specific *KMT2A*-PTD when it detected all the others, but based on the result with the alternative pipeline of analysis, it was not because of a low frequency (VAF = 0.47) or the small size (>49 kb) of the duplication.

Less common *KMT2A*-PTD variants were also properly detected as insertions in our samples: repeats of exons 3 to 6 (three copies) in case 14H172 and exons 5 to 6 (two copies) in case 15H071 (Figure 3d). The latter had the smallest anomaly detected with an insertion size of just 3.42 kb.

In all cases analyzed, the repeat pattern of the DLE-1 sites was consistent with the exons expected to be involved in the variant, but OGM by itself could not identify what specific exons were involved in the repeat. However, in the last sample analyzed where three types of junctions have been detected in the RNA analysis, a likely corresponding variant could be identified for each of them in the DNA (18H194, Figure 3e). As there was no evidence of copy gain for this locus and based on the variant allele frequencies (VAFs), the OGM data were consistent with the presence of four alleles (one wild type and three variants) present in two distinct clones: one major clone containing a *KMT2A*-PTD of exons 3 to 4 and the wild-type allele and one minor clone containing *KMT2A*-PTDs of exons 2 to 10 and exons 2 to 16.

**Figure 4:**
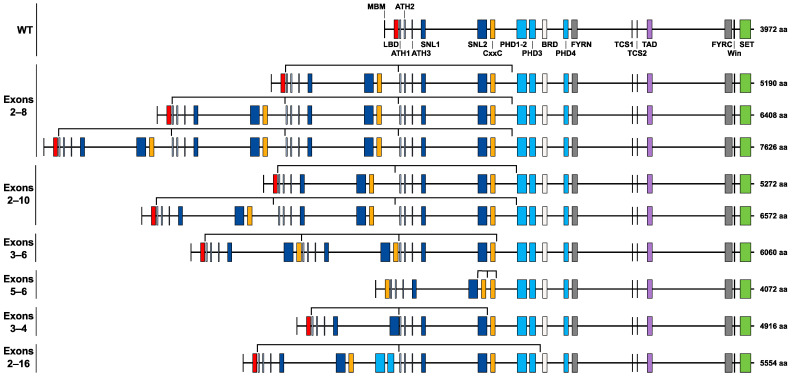
Putative KMT2A-PTD proteins expressed from the DNA variants characterized in this study. Colored rectangles represent protein domains (adapted from [35]). Black lines above the protein maps indicate the repeated regions. MBM: menin-binding motif; LBD: LEDGF-binding domain; ATH1-2-3: AT-hooks; SNL1-2: nuclear localization signals; CxxC: zinc finger-CxxC domain; PHD1-2-3-4: plant homology domains; BRD: bromodomain; FYRN: FY-rich N-terminal domain; TCS1-2: taspase1 cleavage sites; TAD: transactivator domain; FYRC: FY-rich C-terminal domain. Win: WDR5 interaction motif; SET: SET domain (H3K4 methyltransferase activity).

The *KMT2A*-PTD variants identified in our 20 samples were all in-frame variants and when considering both the nature and number of repeats, our analysis identified 9 distinct variants that could potentially generate 9 types of KMT2A-PTD proteins (Figure 4). A change in the number of repeats of the duplicated fragment would be expected to have significant consequences on the resulting protein, assuming that all of them are maintained in the mature transcript of the gene.

## 4. Discussion

The identification of *KMT2A*-r is very important for the management of AML patients. The analysis of clinical samples by OGM has demonstrated that both *KMT2A* fusion genes and *KMT2A*-PTDs can be detected using this technique [21,22,23,24,25,26]. In this work, we used all the information available for AML samples with known *KMT2A*-r status, including the karyotype, *KMT2A* break-apart FISH and transcript analyses, to better characterize the genomic events associated with cryptic or hard-to-characterize *KMT2A* rearrangements using OGM and to evaluate the limits of this technology for the study of these genetic alterations.

All *KMT2A* fusions that were investigated in this report were detected as translocations with the format t(v;11q23.3)(v;118479068), where the nucleotide coordinate in chromosome band 11q23.3 represents the DLE-1 site upstream of the BCR1 of *KMT2A*. The events involved in the formation of the fusion genes were reconstituted by the investigation of co-occurring anomalies and provided an explanation for the atypical *KMT2A* FISH results in most of the analyzed samples. The 3’ fusion partners identified were concordant with the existing transcript fusion data available. There were, however, two cases without transcriptomics data where some uncertainty remained on the fusion partner following OGM analysis: a *KMT2A*::*MYCBP* fusion that had never been described before and a *KMT2A*::*CBL* fusion where the DLE-1 pattern of the *CBL* gene has not been identified by the algorithm. However, in both cases, the translocation identifying the fusion gene was associated with the nearest DLE-1 site (chr11:118479068) upstream of the BCR1, as observed for the other samples with *KMT2A* fusions. Our hypothesis on the nature of the 3′ partner based on the available information was confirmed by Sanger sequencing in both cases but, even without such a confirmation, any translocation identified by OGM at this specific location should be considered a strong indication of the presence of a *KMT2A* fusion.

With the exception of *KMT2A*::*MLLT3*, the 2022 ELN risk classification includes all other *KMT2A* fusions in a single genetic entity with poor prognosis [2]. Although these fusions share a similar transcriptomic signature [28], the identification of the partner gene could reveal subcategories of *KMT2A* fusions in regard to prognosis and/or response to targeted therapies. In line with this, a recent publication of the AUGMENT-101 trial (ClinicalTrials.gov identifier: NCT04065399) that evaluates the menin inhibitor Revumenib in *KMT2A*-rearranged relapsed/refractory AML [9] reported the response rate of this compound in a limited number of patients with the most frequent *KMT2A* translocations. Increasing the number of fully characterized cases with different *KMT2A*-r could help to refine the prognostication and prediction of response to these novel precision therapies. Our results also revealed that a reciprocal 5′gene partner::3′*KMT2A* fusion gene occurring in the context of some atypical fusions can be identified by OGM. With the demonstration of potential oncogenic function for specific reciprocal KMT2A fusion proteins [36,37], identifying the presence of such events should help investigate their involvement in malignant transformation.

Our analysis of 20 cases of *KMT2A*-PTDs identified a surprisingly large diversity of variants, given the limited number of samples. The presence of aberrant *KMT2A* exon-exon junctions had already identified repeats in the N-terminal part of the gene, but the OGM analysis we present in this work showed that identical aberrant junction results could reveal several types of variants presenting a different number of copies of the repeated exons. When combining the nature of the exons involved and the number of repeats identified, we observed 9 distinct in-frame variants in our cohort, including 3 variants identified in the same sample. These variants would be expected to generate 9 distinct proteins with different N-terminal regions. The mechanism of action of the menin inhibitors on KMT2A fusion proteins requires an interaction between the fusion protein and menin to maintain the aberrant expression of the *HOX* and *MEIS* genes characteristic of such rearrangements. *KMT2A*-PTDs are defined by a transcriptomic signature different from *KMT2A* fusions but still constitute a group of AML with the abnormal expression of *HOX* genes [28]. Given that all the putative proteins resulting from the *KMT2A*-PTD variants maintain the original menin-binding motif (MBM), it is plausible that these rearrangements might be targetable by menin inhibitors. To address this question, patients with *KMT2A*-PTD AML are now included in some ongoing clinical trials, notably for the menin inhibitor JNJ-75276617 (ClinicalTrials.gov identifier: NCT04811560) [38].

It is unclear at this stage whether the nature of the *KMT2A*-PTD rearrangement could affect prognosis and/or malignant transformation, and further studies are needed to address these questions. The presence of several distinct repeat patterns and of an absence-of-heterozygosity in some cases is consistent with the possibility of the formation and/or selection of *KMT2A*-PTDs as a multistep process. Our observations are reminiscent of a phenomenon described in a recent study based on a DNA NGS analysis of *KMT2A*-PTDs, showing a progressive increase in the complexity and copy number of *KMT2A*-PTD variants with time, notably during the progression from MDS to AML [18]. The fact that most samples in our study and in published cases display only one type of variant could indicate that the process is mostly completed by the time the AML is diagnosed. Longitudinal OGM studies of patient samples will determine if *KMT2A*-PTD variants can evolve in that fashion.

Our findings suggest that OGM could improve the identification of *KMT2A*-r and other cryptic and/or complex rearrangements. This is in line with the results of other studies where OGM analysis revealed variants of prognostic and/or therapeutic value in myelodysplastic syndromes and acute myeloid leukemias that had been undetected by standard-of-care methods [22,23,24,39]. Moreover, a multicenter study has shown that OGM simplifies the investigation algorithm for hematological cancers by studying the whole genome upfront in an unbiased manner, with a turnaround time shorter than standard cytogenetics [39]. Importantly, guidelines for the implementation of OGM in clinical genetic laboratories were recently published [25].

## 5. Conclusions

*KMT2A*-r have major implications for the diagnosis, prognosis and treatment of AML patients. Considering the vast heterogeneity of *KMT2A*-r, the analysis of large native DNA molecules allowed by OGM promises to become an important tool in identifying and understanding the multiple subtypes of *KMT2A*-r and their clinical relevance. However, OGM results might benefit from a complementary transcript analysis for a full characterization of detected variants.

## Figures and Tables

**Table 1:** Sample characteristics.

Characteristics	*KMT2A* Fusion *n* = 12	*KMT2A*-PTD *n* = 20 ^1^	No *KMT2A* Anomaly *n* = 6
Age—median (range)	50 (25–71)	61.5 (29–76)	52 (21–71)
Sex (male)—*n* (%)	7 (58)	14 (70)	3 (50)
Time point			
Diagnosis—*n* (%)	11 (92)	18 (90)	6 (100)
Relapse/refractory—*n* (%)	1 (8)	2 (10)	0
AML type			
De novo—*n* (%)	11 (92)	16 (80)	6 (100)
Post-MDS—*n* (%)	0	2 (10)	0
Post-cytotoxic—*n* (%)	1 (8)	2 (10)	0
Normal karyotype—*n* (%)	2 (17)	12 (60)	6 (100)
*FLT3*-ITD mutation—*n* (%)	0	12 (60)	4 (67)
*NPM1* mutation ^2^—*n* (%)	0/8	0/19	5 (83)
*KMT2A* fusion (transcript) ^2^—*n* (%)	9/9 (100)	0	0
*KMT2A*-PTD (transcript) ^2^—*n* (%)	0/9	20 (100)	0

^1^ Samples from 19 patients. ^2^ Not available for all samples. ITD: internal tandem duplication. MDS: myelodysplastic syndrome. PTD: partial tandem duplication.

**Table 2:** Description of the events identified by OGM analysis in 12 cryptic/hard-to-characterize *KMT2A* fusion cases.

Sample	*KMT2A* Break-Apart FISH Signals	*KMT2A* Locus	Fusion Partner Gene Locus	Associated Event(s)	Figure
09H102	2 fusions	insertion of 3′*MLLT10* in the *KMT2A* BCR1	3′*MLLT10* (1.78 Mb) deletion		Figure S1
11H095	2 fusions	duplication of 5′*KMT2A* with insertion of 3′*MLLT10* copy gain in the *KMT2A* BCR1	3′*MLLT10* copy gain (126 kb)		Figure S2
22H021	1 fusion with separate 5′ and 3′ signals	reciprocal t(10;11)(p12.31;q23.3)/*KMT2A*::*MLLT10*		reciprocal t(5;10)(q15;p14)	Figure S3
21H024	1 fusion with separate 5′ and 3′ signals and extra 3′ signal	reciprocal t(1;11)(p34.3;q23.3)/*KMT2A*::*MYCBP*		extra 3′ signal due to 11q copy gain downstream of *KMT2A*	Figure S4
02H033	2 fusions with extra 5′ signal on der(1)	5′*KMT2A* copy gain (1.0 Mb)	5′*ENAH* deletion (326 kb) in chr1 with insertion of 5’*KMT2A* copy gain		Figure S5
06H073	2 fusions with extra 5′ signal on der(10)	5′*KMT2A* copy gain (1.0 Mb)	5′*MLLT10* deletion (329 kb) with insertion of 5’*KMT2A* copy gain		Figure S6
18H072	2 fusions (1 on der(X)) with extra 5′ signal on der(12)	inv(11) at *KMT2A* BCR1 (176 kb) combined to t(X;11)(q24;q23.3)/*KMT2A*::*SEPTIN6* resulting in fusion signal on der(X)		extra 5′ signal likely due to a second t(X;11) rearrangement affecting the sequences targeted by the probe upstream of *KMT2A*	Figure S7
07H160	1 fusion with 5′ signal and loss of 3′ signal	3′*KMT2A* deletion (231 kb) and insertion of deleted 3’*MLLT10*	3′*MLLT10* (183 kb) deletion		Figure S8
10H031	1 fusion with 5′ signal and loss of 3′ signal	3′*KMT2A* deletion (243 kb) and insertion of 3′*AFDN* copy gain	3′*AFDN* copy gain (178 kb)		Figure S9
05H128	1 fusion with 5′ signal on der(10) and 3′ signal on der(11)	5′*KMT2A* (28.9 Mb) deletion	5′*MLLT10* deletion (7.8 Mb) with insertion of deleted 5’*KMT2A* on chr10		Figure S10
06H077	1 fusion with 5′ signal on der(11), 5′ signal on der(10) and loss of 3′ signal	deletion of *KMT2A* (774 kb) except small 5′*KMT2A* 107 kb region inserted in rearranged *MLLT10*	inv(10) with breakpoint in *MLLT10* (9.1 Mb) and insertion of small 5′*KMT2A*	5′ signal on der(11) due to undeleted 5′FISH probe targeting a sequence upstream of *KMT2A*	Figure S11
07H114	1 fusion with 5′ and 3′ signals both on der(X)	inv(11)(q23.3q23.3)/*KMT2A*::*CBL*		*KMT2A*::*CBL* fusion gene translocated to *USP9X* locus on Xp11.4	Figure 2 and Figure S12

## Data Availability

RNAseq data used for the transcript analyses are available in a publicly accessible repository for 23 of the samples (GEO accession numbers are indicated in Table S2). Optical genome mapping data are contained within the article or Supplementary Materials. Additional data are available upon request to the corresponding author.

## Supplementary Materials

The following supporting information can be downloaded at: https://www.mdpi.com/article/10.3390/cancers16244171/s1, Figure S1: *KMT2A*::*MLLT10* fusion rearrangement in case 09H102; Figure S2: *KMT2A*::*MLLT10* fusion rearrangement in case 11H095; Figure S3: *KMT2A*::*MLLT10* reciprocal fusion rearrangements in case 22H021; Figure S4: *KMT2A*::*MYCBP* reciprocal fusion rearrangements in case 21H024; Figure S5: *KMT2A*::*ENAH* fusion rearrangement in case 02H033; Figure S6: *KMT2A*::*MLLT10* fusion rearrangement in case 06H073; Figure S7: *KMT2A*::*SEPTIN6* fusion rearrangement in case 18H072; Figure S8: *KMT2A*::*MLLT10* fusion rearrangement in case 07H160; Figure S9: *KMT2A*::*AFDN* fusion rearrangement in case 10H031; Figure S10: *KMT2A*::*MLLT10* fusion rearrangement in case 05H128; Figure S11: *KMT2A*::*MLLT10* fusion rearrangement in case 06H077. Figure S12: *KMT2A*::*CBL* fusion rearrangement in case 07H114; Figure S13: Circos plots of control samples with no *KMT2A* anomaly; Figure S14: Circos plots of *KMT2A*-PTD cases; Figure S15: *RUNX1* variants in *KMT2A*-PTD cases; Figure S16: Other *KMT2A*-PTD variants; Table S1: Additional sample information; Table S2: *KMT2A* anomalies identified by transcript analyses; Table S3: Default parameters for bioanalysis with Rare Variant Pipeline and VIA for AML hg38 sample type; Table S4: Quality metrics for the Rare Variant Pipeline and VIA analyses; Table S5: List of validated OGM variant calls for Rare Variant Pipeline and VIA analyses; Table S6: Comparison of validated variants for the same patient at time of diagnosis (06H146) and at the refractory stage (07H152); Table S7: Variant calls at *KMT2A* locus for cases reanalyzed with Guided Assembly—Low Allele Frequency pipeline of analysis and VIA.
