# Supplementary material for: Unveiling the Complexity of KMT2A Rearrangements in Acute Myeloid Leukemias with Optical Genome Mapping
Source: Cancers (Basel). 2024 Dec 14;16(24):4171. doi: 10.3390/cancers16244171 (PMC11674939; doi:10.3390/cancers16244171)
Supplement: Supplementary file 1 [file cancers-16-04171-s001.zip › Supplementary-Materials_proofed_final.pdf]

Supplementary Materials

Unveiling the Complexity of *KMT2A* Rearrangements in Acute Myeloid Leukemias with Optical Genome Mapping

Sandrine A. Lacoste [1], Vanessa Gagnon [1], François Béliveau [1], Sylvie Lavallée [1], Vanessa Collin [1,2] and Josée Hébert [1,2,3,4*]

[1] Quebec Leukemia Cell Bank, Maisonneuve-Rosemont Hospital, Montréal, QC H1T 2M4, Canada; sandrine.lacoste.cemtl@ssss.gouv.qc.ca; vanessa.gagnon2@hotmail.fr; francois.beliveau.cemtl@ssss.gouv.qc.ca; slavallee.hmr@ssss.gouv.qc.ca; vanessa.collin.cemtl@ssss.gouv.qc.ca; josee.hebert@umontreal.ca.

[2] Cytogenetics Laboratory, Maisonneuve-Rosemont Hospital, Montréal, QC H1T 2M4, Canada.

[3] Division of Hematology-Oncology and Cellular therapy, Maisonneuve-Rosemont Hospital, Montréal, QC H1T 2M4, Canada.

[4] Department of Medicine, Faculty of Medicine, Université de Montréal, Montréal, QC H3T 1J4, Canada.

* Correspondence: josee.hebert@umontreal.ca; Tel.: +1-514-252-3404

1. Supplementary Tables (Excel files)

Table S1. Additional sample information.

Table S2. *KMT2A* anomalies identified by transcript analyses.

Table S3. Default parameters for bioanalysis with Rare Variant Pipeline and VIA for AML hg38 sample type.

Table S4. Quality metrics for the Rare Variant Pipeline and VIA analyses.

Table S5. List of validated OGM variant calls for Rare Variant Pipeline and VIA analyses.

Table S6. Comparison of validated variants for the same patient at time of diagnosis (06H146) and at the refractory stage (07H152).

Table S7. Variant calls at *KMT2A* locus for cases reanalyzed with Guided Assembly – Low Allele Frequency pipeline of analysis and VIA.

2. Supplementary Figures

Figure S1. *KMT2A*::*MLLT10* fusion rearrangement in case 09H102. 3

Figure S2. *KMT2A*::*MLLT10* fusion rearrangement in case 11H095. 4

Figure S3. *KMT2A*::*MLLT10* reciprocal fusion rearrangements in case 22H021. 5

Figure S4. *KMT2A*::*MYCBP* reciprocal fusion rearrangements in case 21H024. 6

Figure S5. *KMT2A*::*ENAH* fusion rearrangement in case 02H033. 7

Figure S6. *KMT2A*::*MLLT10* fusion rearrangement in case 06H073. 8

Figure S7. *KMT2A*::*SEPTIN6* fusion rearrangement in case 18H072. 9

Figure S8. *KMT2A*::*MLLT10* fusion rearrangement in case 07H160. 10

Figure S9. *KMT2A*::*AFDN* fusion rearrangement in case 10H031. 11

Figure S10. *KMT2A*::*MLLT10* fusion rearrangement in case 05H128. 12

Figure S11. *KMT2A*::*MLLT10* fusion rearrangement in case 06H077. 13

Figure S12. *KMT2A*::*CBL* fusion rearrangement in case 07H114. 14

Figure S13. Circos plots of control samples with no *KMT2A* anomaly. 15

Figure S14. Circos plots of *KMT2A*-PTD cases. 16-17

Figure S15. *RUNX1* variants in *KMT2A*-PTD cases. 18

Figure S16. Other *KMT2A*-PTD variants analyzed. 19

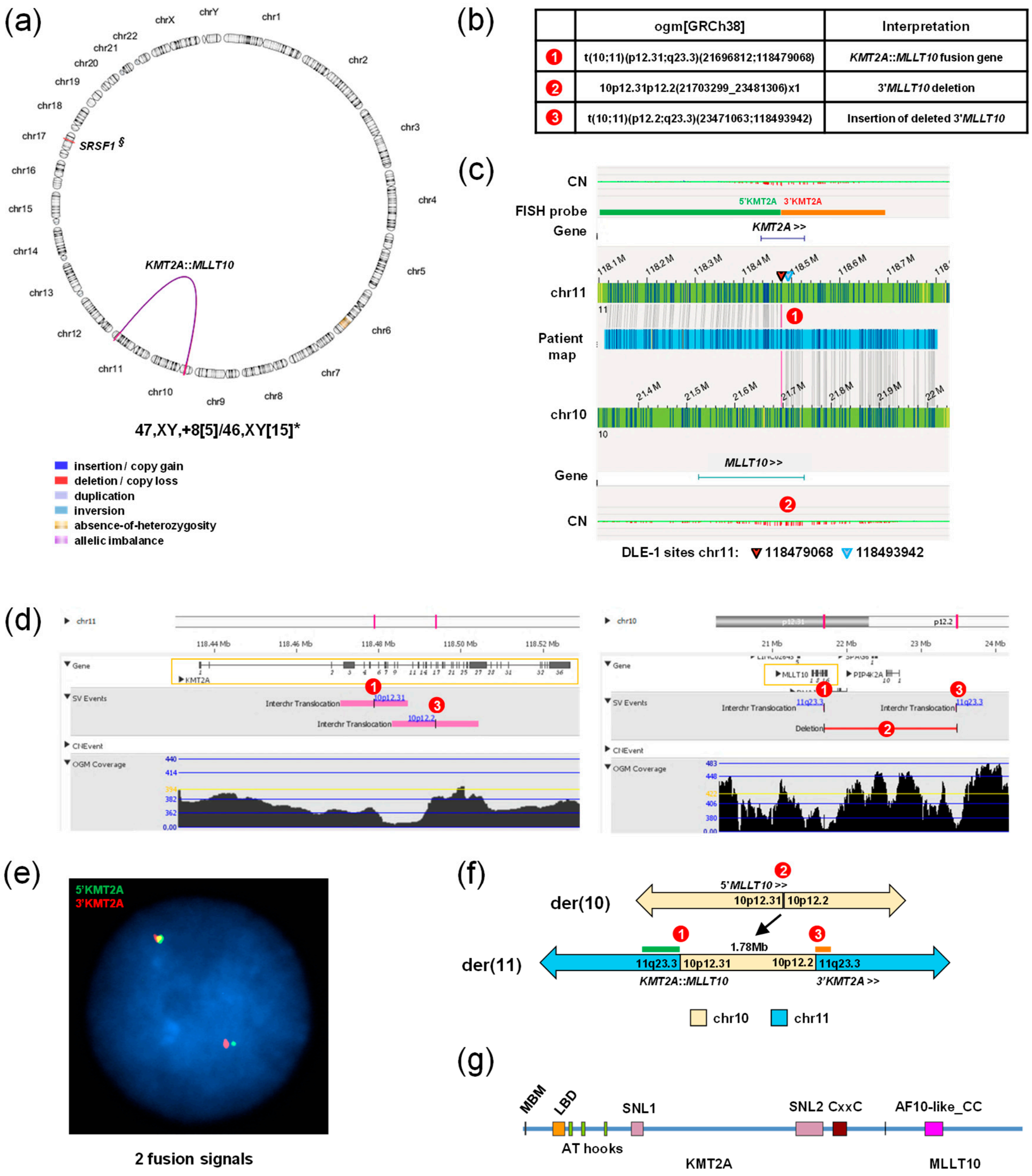

Figure S1. *KMT2A*::*MLLT10* fusion rearrangement in case 09H102.

(a) Circos plot and karyotype result. Chromosomes are displayed in a circular pattern with the location of variant calls indicated by colored markers depending on the type of variant. Translocation calls are shown as colored arcs inside the circos plot. (b) Validated variants associated with the *KMT2A*-r and their interpretation. (c) *KMT2A*::*MLLT10* fusion variant in Access. (d) *KMT2A* and *MLLT10* loci in VIA. (e) *KMT2A* break-apart interphase FISH analysis. (f) Partial putative der(10) and der(11) based on variants identified by OGM (green/orange rectangles indicate FISH break-apart probe approximate location). (g) Putative KMT2A-MLLT10 fusion protein based on fusion transcript information. MBM: menin-binding motif; LBD: LEDGF-binding domain; SNL1-2: nuclear-localization signals; CxxC: zinc finger-CxxC domain; AF10-like_CC: coiled-coil domain. Numbers in panels c–d and f correspond to OGM variant calls listed in panel b. *Trisomy 8 was confirmed by interphase FISH in 6.5% of cells but was not detected by OGM. § *SRSF1* deletion.

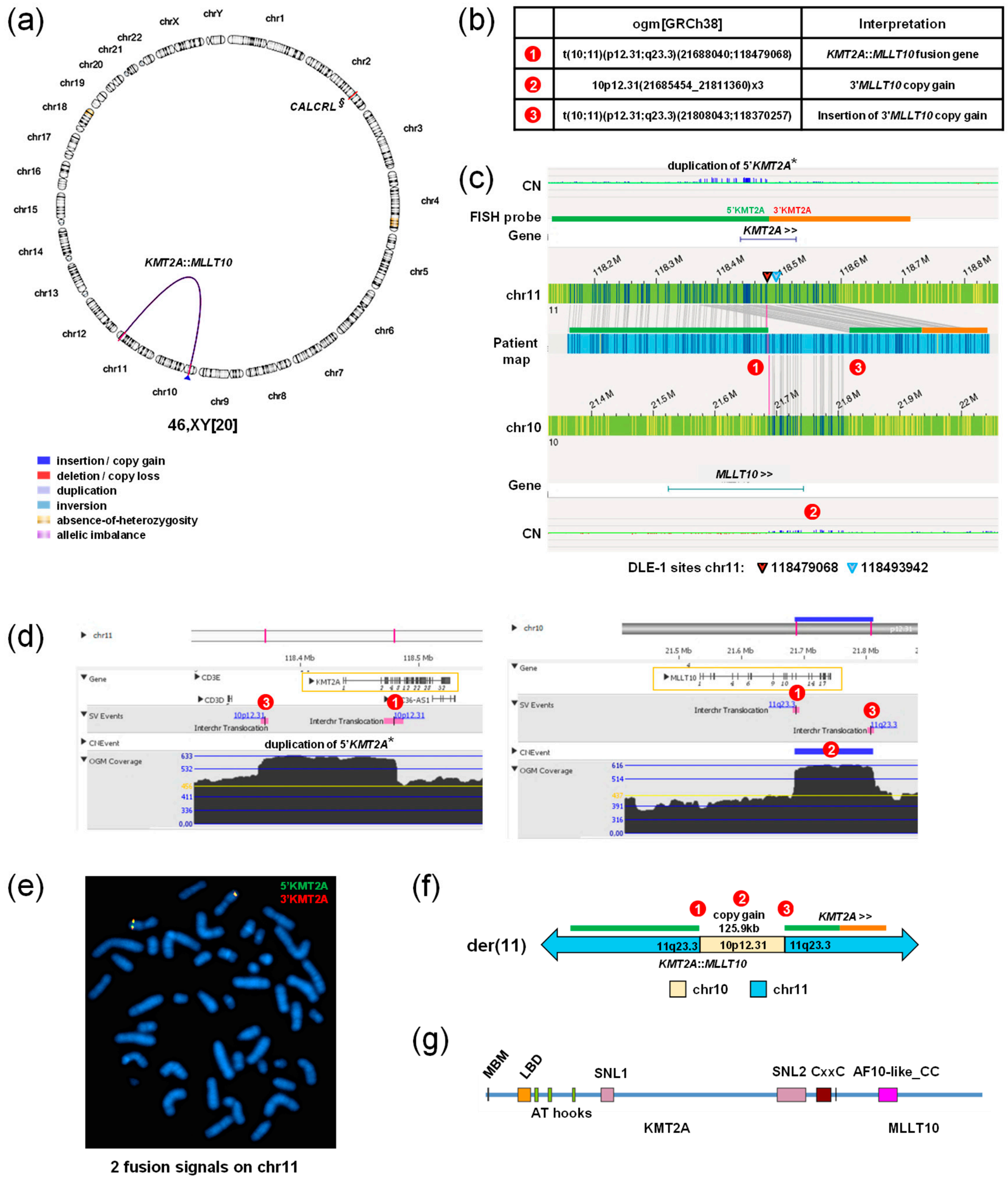

Figure S2. *KMT2A*::*MLLT10* fusion rearrangement in case 11H095.

(a) Circos plot and karyotype result. Chromosomes are displayed in a circular pattern with the location of variant calls indicated by colored markers depending on the type of variant. Translocation calls are shown as colored arcs inside the circos plot. (b) Validated variants associated with the *KMT2A*-r and their interpretation (c) *KMT2A*::*MLLT10* fusion variant in Access. (d) *KMT2A* and *MLLT10* loci in VIA. (e) *KMT2A* break-apart metaphase FISH analysis. (f) Partial putative der(11) based on variants identified by OGM (green/orange rectangles indicate FISH break-apart probe approximate location). (g) Putative KMT2A-MLLT10 fusion protein based on fusion transcript information. MBM: menin-binding motif; LBD: LEDGF-binding domain; SNL1-2: nuclear-localization signals; CxxC: zinc finger-CxxC domain; AF10-like_CC: coiled-coil domain. Numbers in panels c-d-f correspond to OGM variant calls listed in panel b. *duplication of 5′*KMT2A* was not called as a distinct variant by OGM analysis. § *CALCRL* deletion.

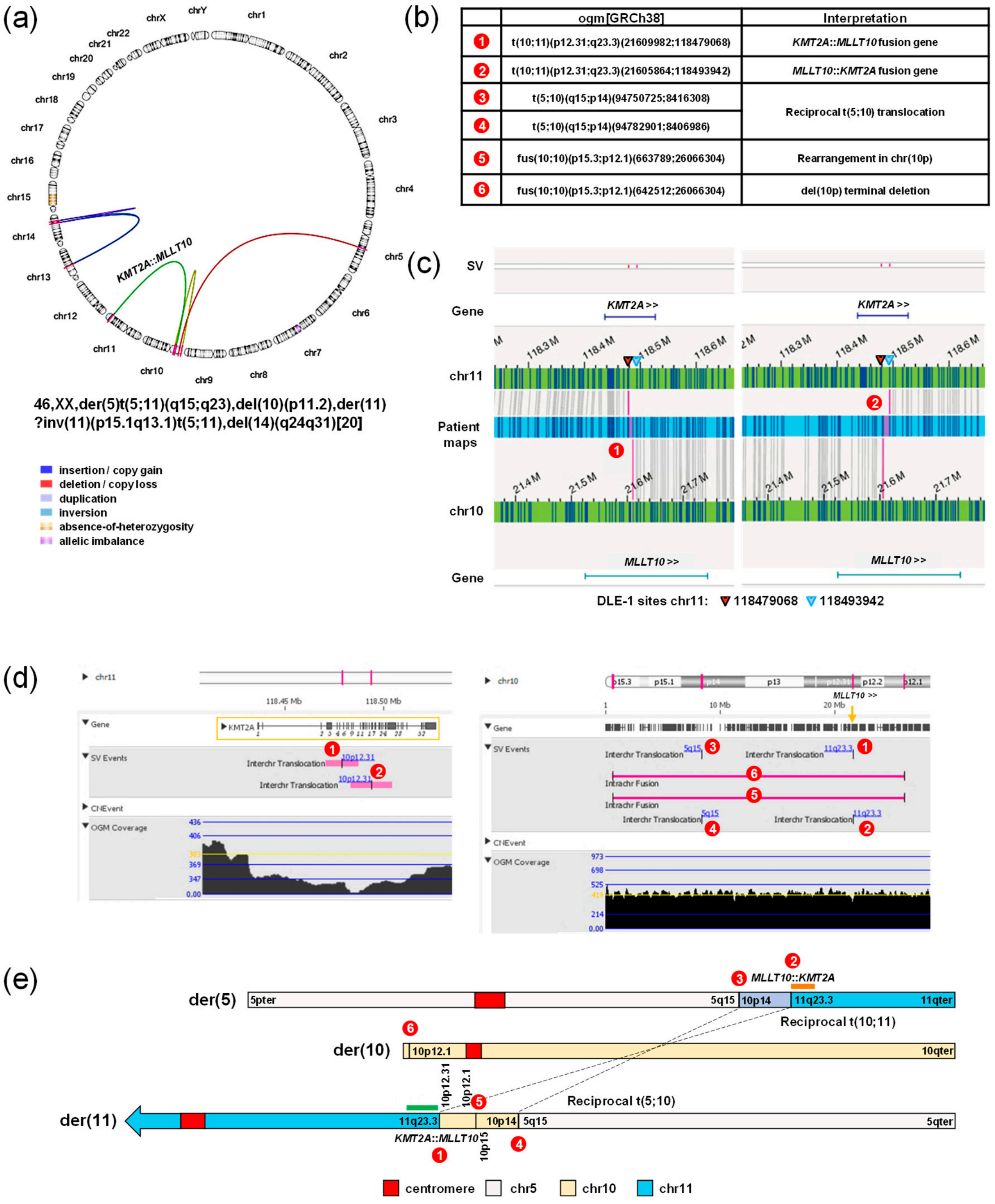

Figure S3. *KMT2A*::*MLLT10* reciprocal fusion rearrangements in case 22H021.

(a) Circos plot and karyotype result. Chromosomes are displayed in a circular pattern with the location of variant calls indicated by colored markers depending on the type of variant. Translocation calls are shown as colored arcs inside the circos plot. (b) Validated variants associated with the *KMT2A*-r and their interpretation. (c) *KMT2A*::*MLLT10* fusion variant and reciprocal in Access. (d) *KMT2A* and *MLLT10* loci in VIA. (e) Putative der(5), der(10) and partial der(11) based on variants identified by OGM (green/orange rectangles indicate FISH break-apart probe approximate location). Numbers in panels c–e correspond to OGM variant calls listed in panel b.

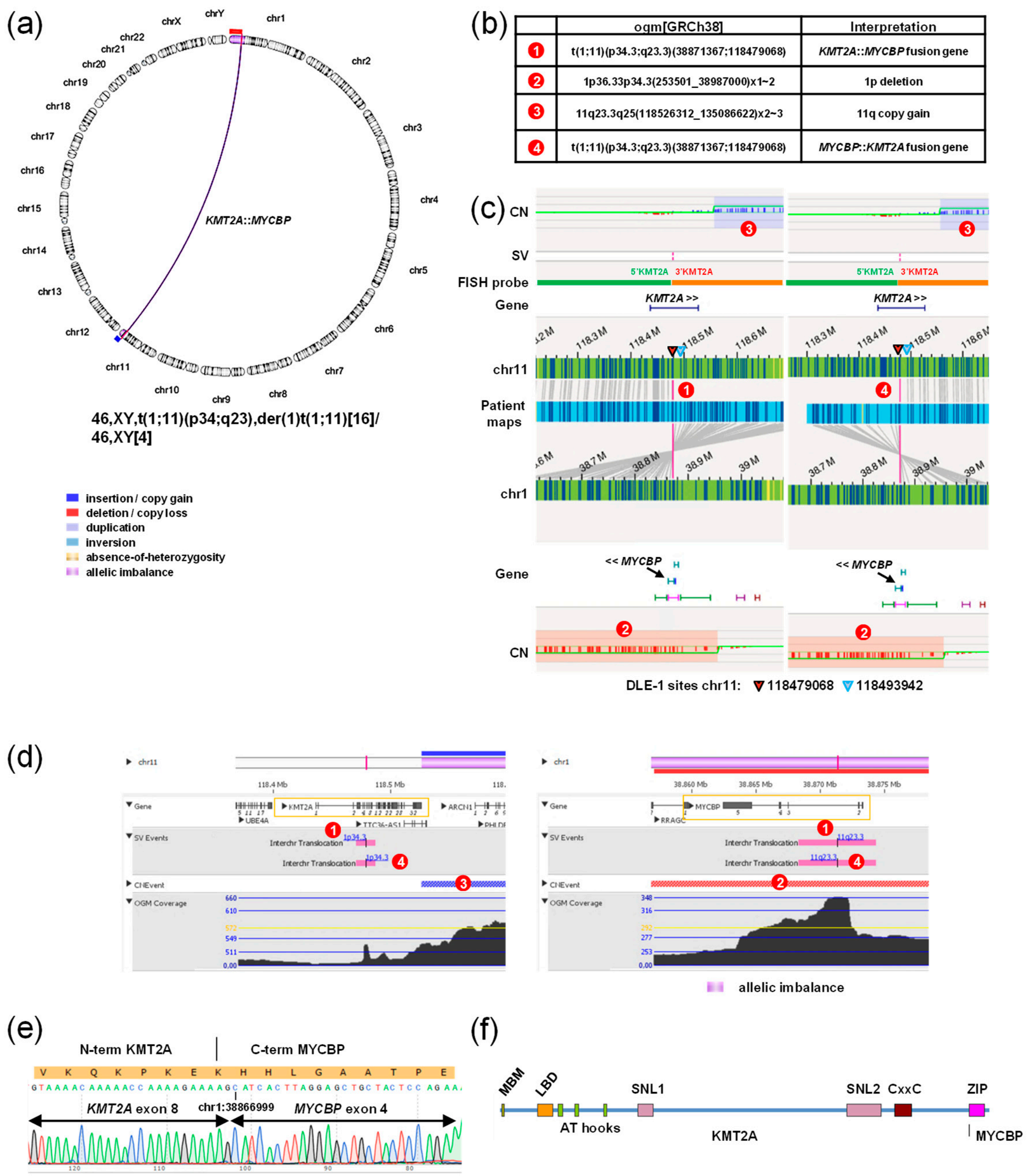

Figure S4. *KMT2A*::*MYCBP* reciprocal fusion rearrangements in case 21H024.

(a) Circos plot and karyotype result. Chromosomes are displayed in a circular pattern with the location of variant calls indicated by colored markers depending on the type of variant. Translocation calls are shown as colored arcs inside the circos plot. (b) Validated variants associated with the *KMT2A*-r and their interpretation. (c) *KMT2A*::*MYCBP* fusion variant and reciprocal in Access. (d) *KMT2A* and *MYCBP* loci in VIA. (e) Sanger sequencing identifying fusion gene junction in RNA transcript. (f) Putative KMT2A-MYCBP fusion protein based on transcript sequencing information. MBM: menin-binding motif; LBD: LEDGF-binding domain; SNL1-2: nuclear-localization signals; CxxC: zinc finger-CxxC domain; ZIP: leucine zipper domain (dimerization domain). Numbers in panels c–d correspond to OGM variant calls listed in panel b.

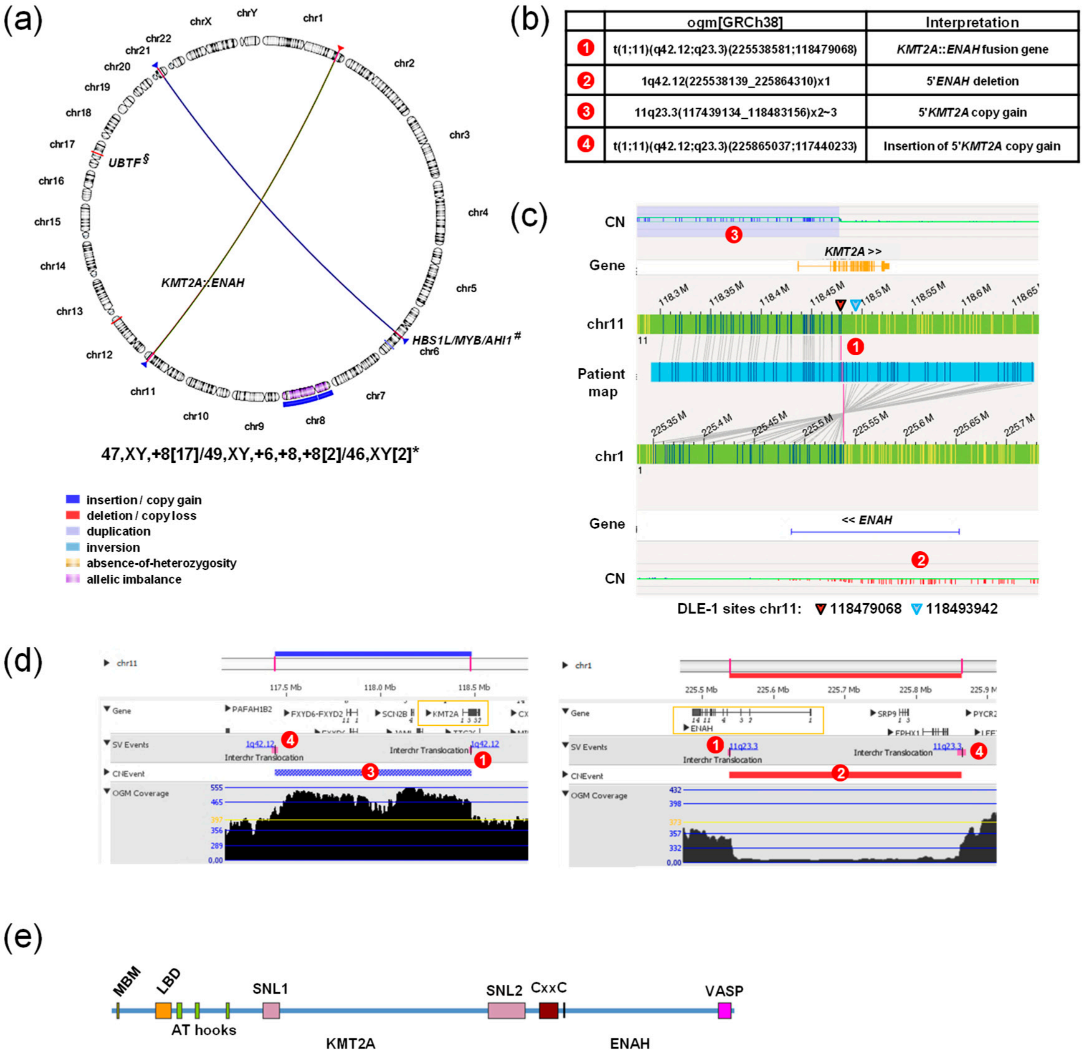

Figure S5. *KMT2A*::*ENAH* fusion rearrangement in case 02H033.

(a) Circos plot and karyotype result. Chromosomes are displayed in a circular pattern with the location of variant calls indicated by colored markers depending on the type of variant. Translocation calls are shown as colored arcs inside the circos plot. (b) Validated variants associated with the *KMT2A*-r and their interpretation. (c) *KMT2A*::*ENAH* fusion variant in Access. (d) *KMT2A* and *ENAH* loci in VIA. (e) Putative KMT2A-ENAH fusion protein based on transcript sequencing information. MBM: menin-binding motif; LBD: LEDGF-binding domain; SNL1-2: nuclear-localization signals; CxxC: zinc finger-CxxC domain; VASP: Vasodilator-stimulated phosphoprotein (tetramerization domain). Numbers in panels c–d correspond to OGM variant calls listed in panel b. *Trisomy 6 (detected in only 2 metaphases) was not detected by OGM. § *UBTF* deletion. # *HBS1L*/*MYB*/*AHI1* locus tandem duplications.

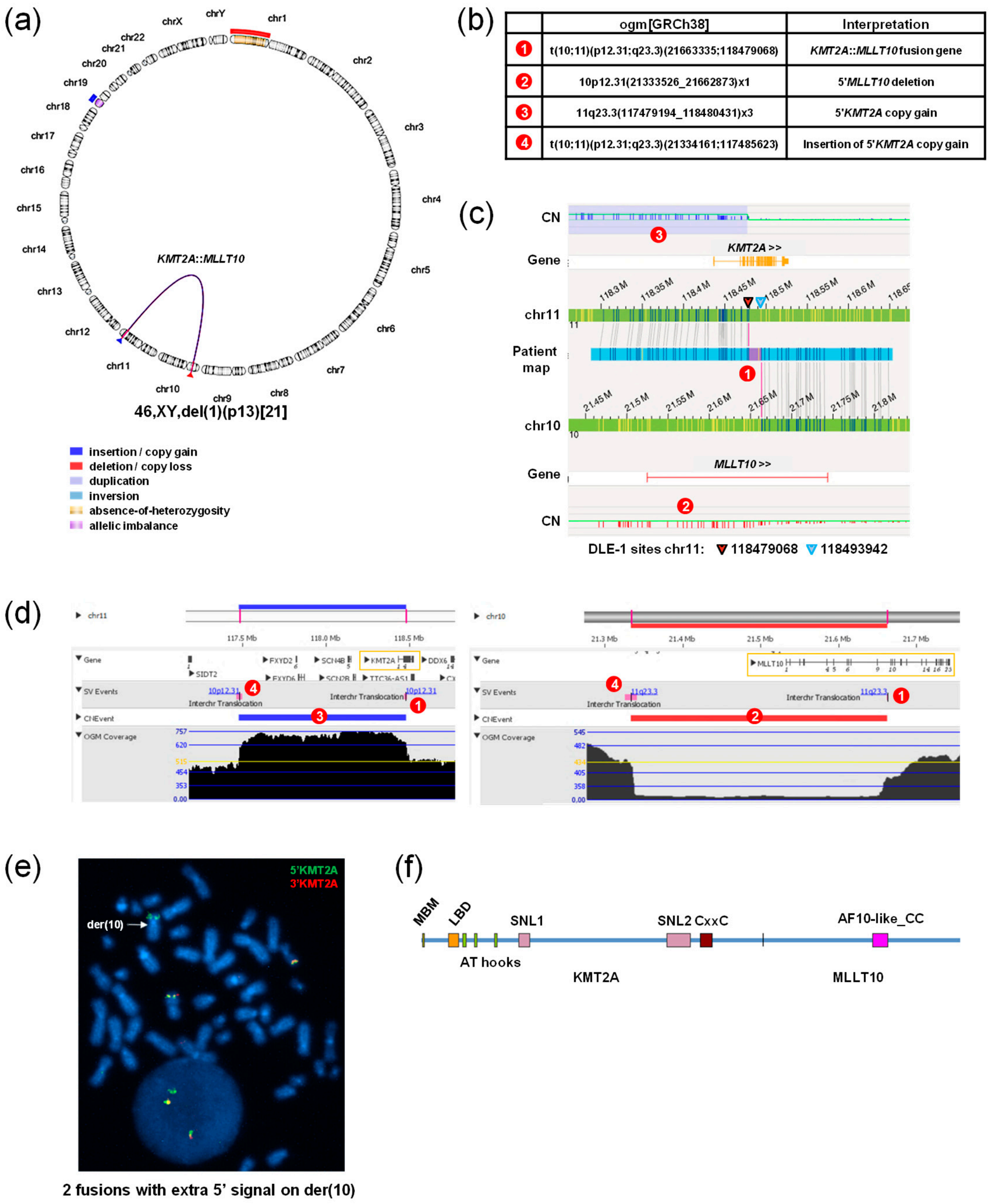

Figure S6. *KMT2A*::*MLLT10* fusion rearrangement in case 06H073.

(a) Circos plot and karyotype result. Chromosomes are displayed in a circular pattern with the location of variant calls indicated by colored markers depending on the type of variant. Translocation calls are shown as colored arcs inside the circos plot. (b) Validated variants associated with the *KMT2A*-r and their interpretation. (c) *KMT2A*::*MLLT10* fusion variant in Access. (d) *KMT2A* and *MLLT10* loci in VIA. (e) *KMT2A* break-apart metaphase FISH analysis. (f) Putative KMT2A-MLLT10 fusion protein based on fusion transcript information. MBM: menin-binding motif; LBD: LEDGF-binding domain; SNL1-2: nuclear-localization signals; CxxC: zinc finger-CxxC domain; AF10-like_CC: coiled-coil domain. Numbers in panels c–d correspond to OGM variant calls listed in panel b.

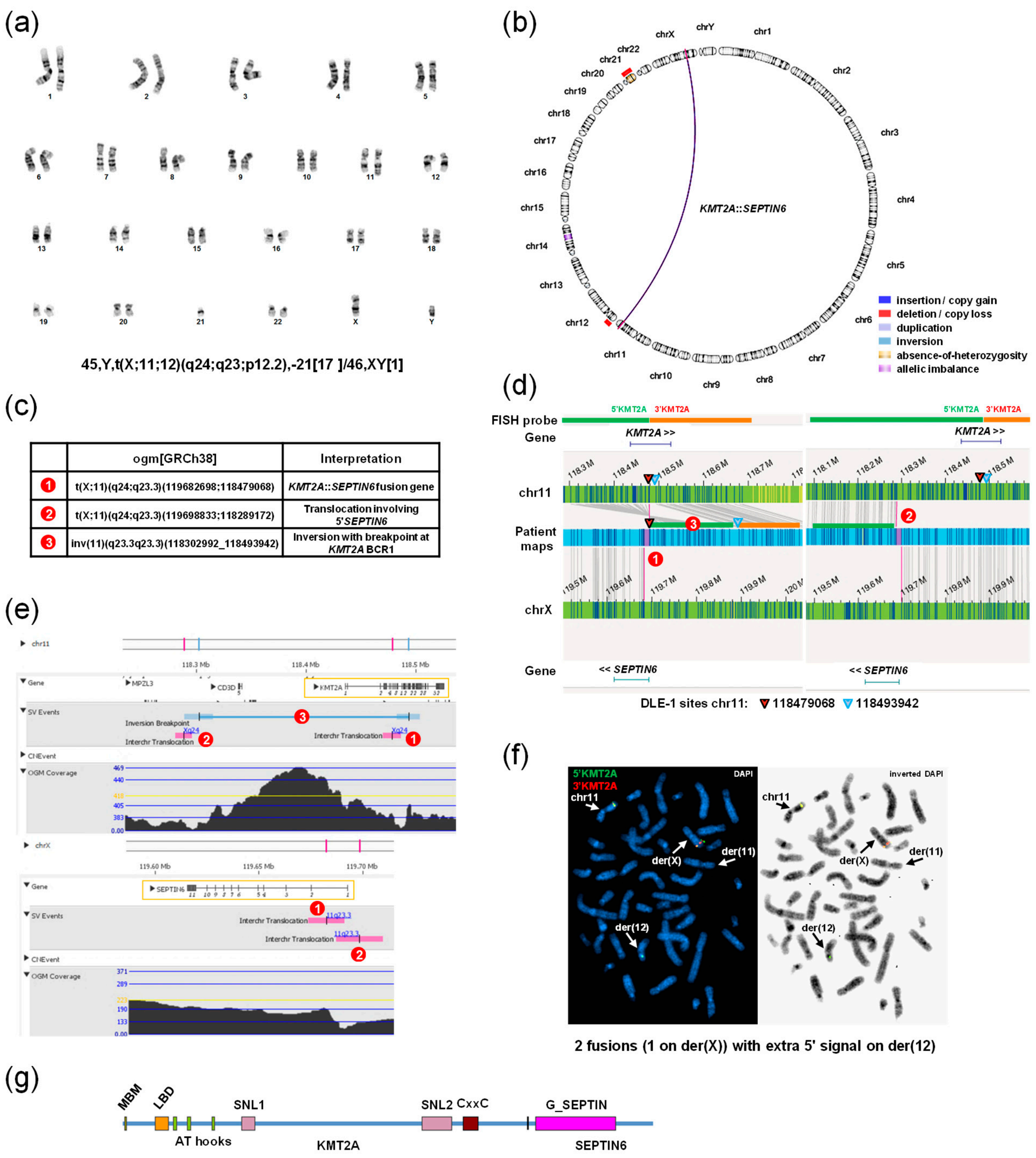

Figure S7. *KMT2A:SEPTIN6* fusion rearrangement in case 18H072.
(a) G-banding karyotype and result. (b) Circos plot. Chromosomes are displayed in a circular pattern with the location of variant calls indicated by colored markers depending on the type of variant. Translocation calls are shown as colored arcs inside the circos plot. (c) Validated variants associated with the *KMT2A*-r and their interpretation. (d) *KMT2A*::*SEPTIN6* fusion variant and second rearrangement breaking the probe targeting sequences upstream of *KMT2A* (possibly responsible for extra 5′ FISH signal). (e) *KMT2A* and *SEPTIN6* loci in VIA. (f) *KMT2A* break-apart metaphase FISH analysis. (g) Putative KMT2A-SEPTIN6 fusion protein based on fusion transcript information. MBM: menin-binding motif; LBD: LEDGF-binding domain; SNL1-2: nuclear-localization signals; CxxC: zinc finger-CxxC domain; G_SEPTIN: Septin-type guanine nucleotide-binding (G) domain. Numbers in panels d–e correspond to OGM variant calls listed in panel c.

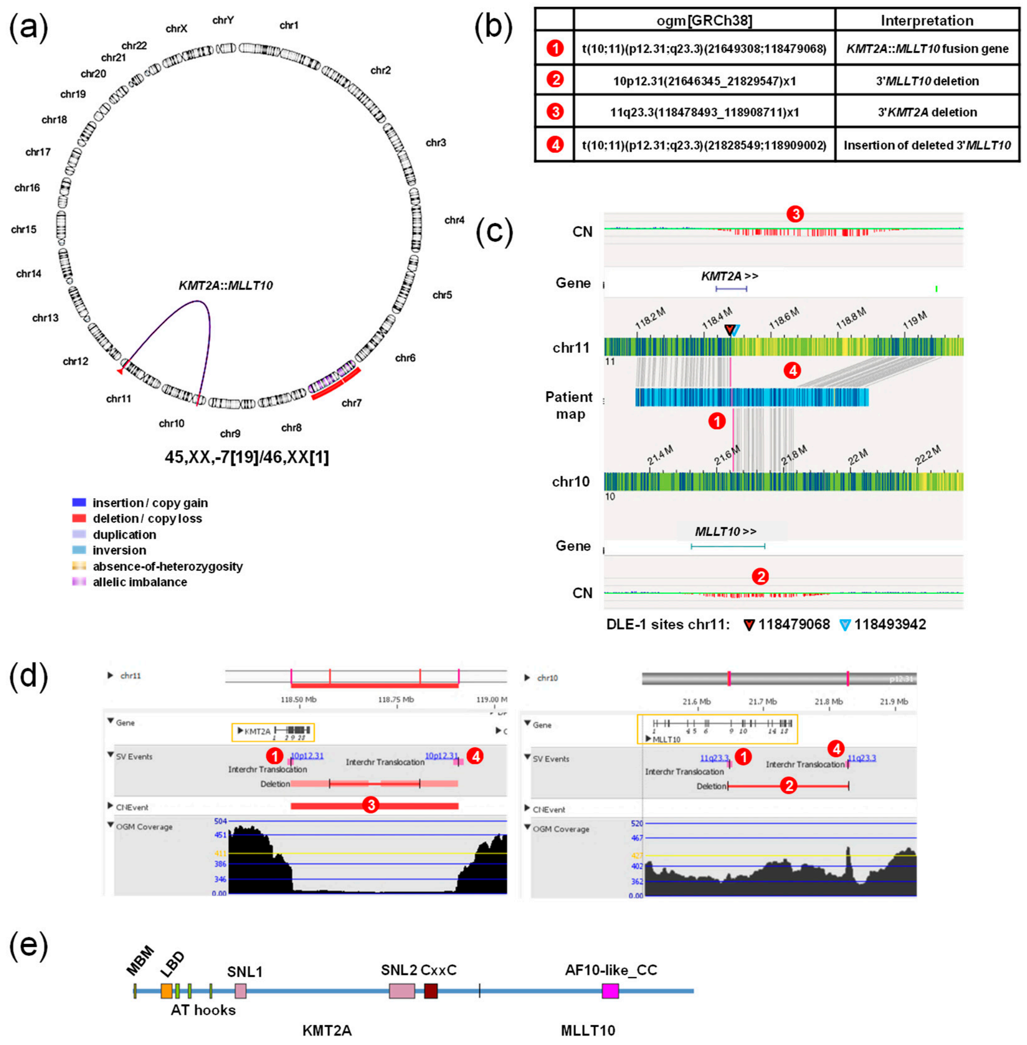

Figure S8. *KMT2A*::*MLLT10* fusion rearrangement in case 07H160.

(a) Circos plot and karyotype result. Chromosomes are displayed in a circular pattern with the location of variant calls indicated by colored markers depending on the type of variant. Translocation calls are shown as colored arcs inside the circos plot. (b) Validated variants associated with the *KMT2A*-r and their interpretation. (c) *KMT2A*::*MLLT10* fusion variant in Access. (d) *KMT2A* and *MLLT10* loci in VIA. (e) Putative KMT2A-MLLT10 fusion protein based on fusion transcript information. MBM: menin-binding motif; LBD: LEDGF-binding domain; SNL1-2: nuclear-localization signals; CxxC: zinc finger-CxxC domain; AF10-like_CC: coiled-coil domain. Numbers in panels c–d correspond to OGM variant calls listed in panel b.

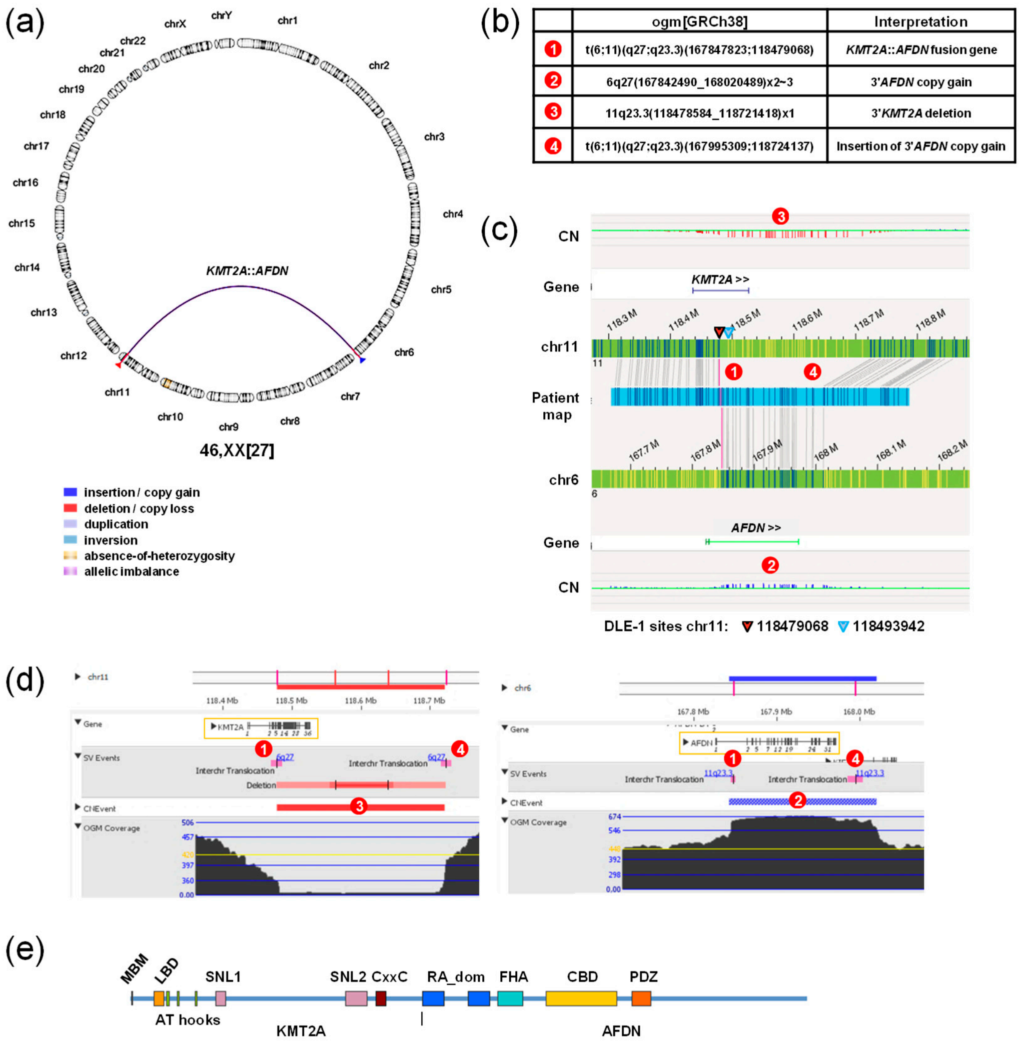

Figure S9: *KMT2A*::*AFDN* fusion rearrangement in case 10H031.
(a) Circos plot and karyotype result. Chromosomes are displayed in a circular pattern with the location of variant calls indicated by colored markers depending on the type of variant. Translocation calls are shown as colored arcs inside the circos plot. (b) Validated variants associated with the *KMT2A*-r and their interpretation. (c) *KMT2A*::*AFDN* fusion variant in Access. (d) *KMT2A* and *AFDN* loci in VIA. (e) Putative KMT2A-AFDN fusion protein based on fusion transcript information. MBM: menin-binding motif; LBD: LEDGF-binding domain; SNL1-2: nuclear-localization signals; CxxC: zinc finger-CxxC domain; RA_dom: Ras-associating domains; FHA: forkhead-associated domain; CBD: cargo binding domain; PDZ: PDZ domain. Numbers in panels c–d correspond to OGM variant calls listed in panel b.

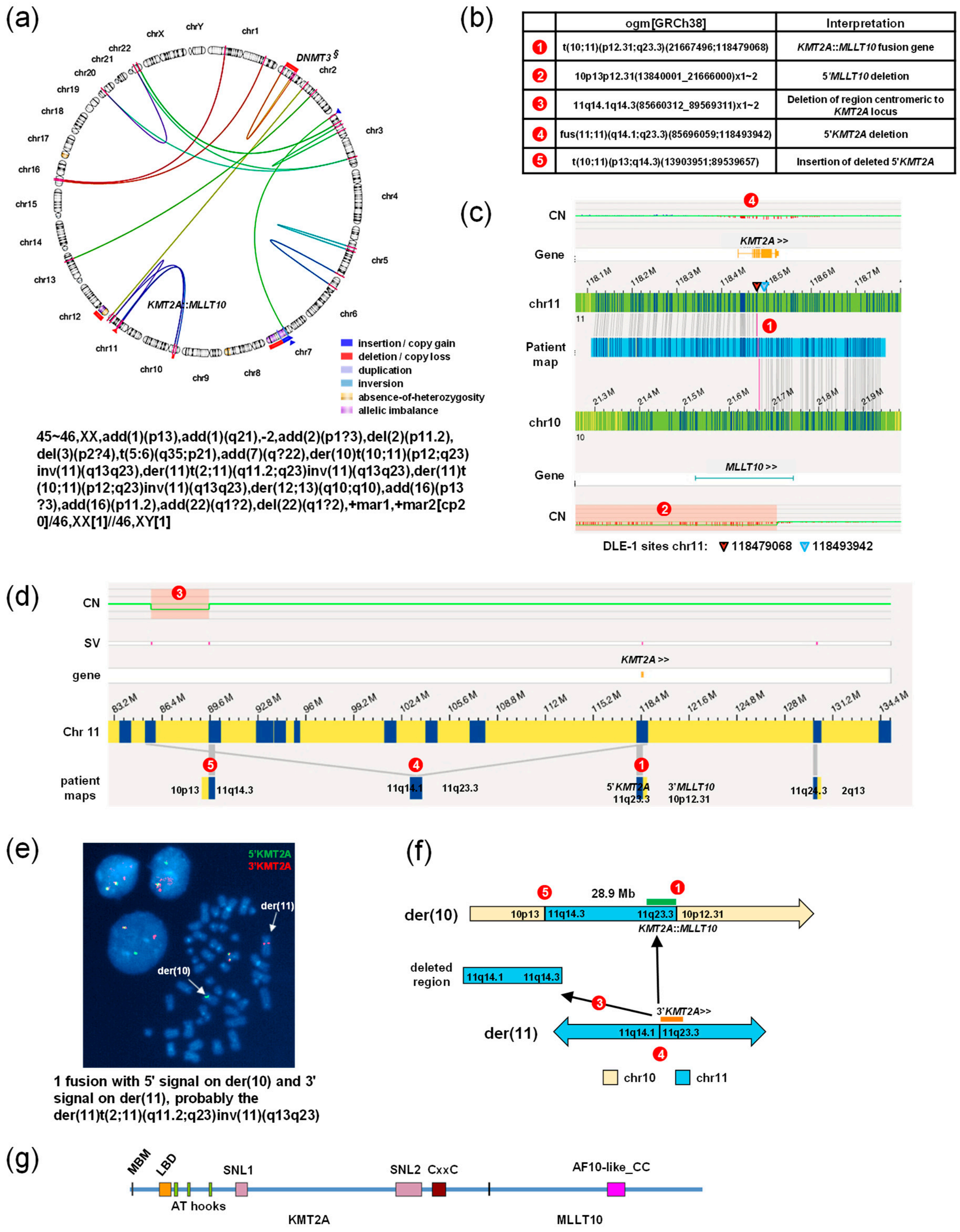

Figure S10. *KMT2A*::*MLLT10* fusion rearrangement in case 05H128.
(a) Circos plot and karyotype result. Chromosomes are displayed in a circular pattern with the location of variant calls indicated by colored markers depending on the type of variant. Translocation calls are shown as colored arcs inside the circos plot. (b) Validated variants associated with the *KMT2A*-r and their interpretation. (c) *KMT2A*::*MLLT10* fusion variant in Access (d) Genome browser view of chromosome 11q14.1-q25 in Access. (e) *KMT2A* break-apart metaphase FISH analysis. (f) Partial putative der(10) and der(11) based on variants identified by OGM (green/orange rectangles indicate FISH break-apart probe approximate location). (g) Putative KMT2A-MLLT10 fusion protein based on fusion transcript information. MBM: menin-binding motif; LBD: LEDGF-binding domain; SNL1-2: nuclear-localization signals; CxxC: zinc finger-CxxC domain; AF10-like_CC: coiled-coil domain. Numbers in panels c–d and f correspond to OGM variant calls listed in panel b. § *DNMT3A* copy loss.

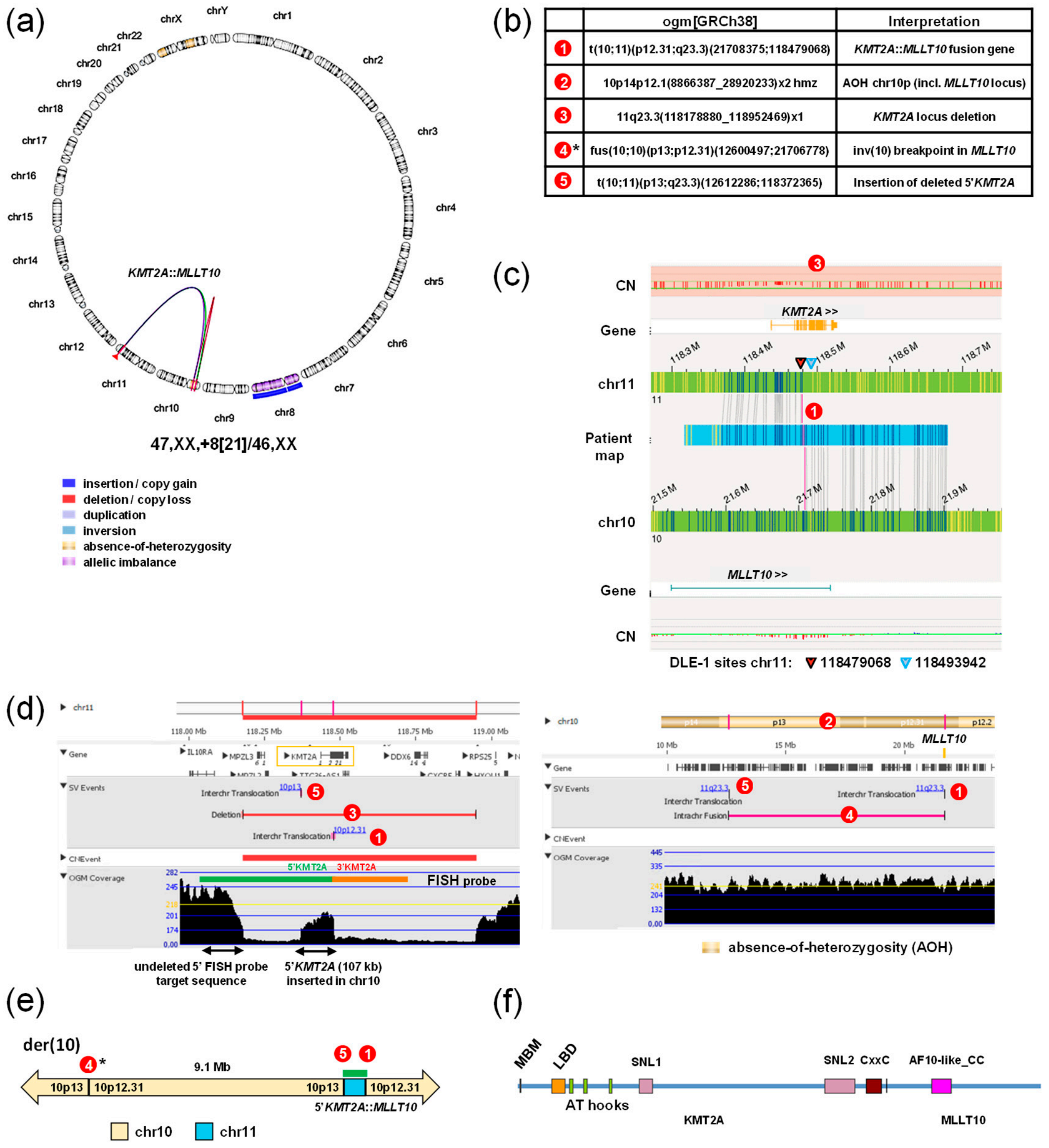

Figure S11. *KMT2A*::*MLLT10* fusion rearrangement in case 06H077.

(a) Circos plot and karyotype result. Chromosomes are displayed in a circular pattern with the location of variant calls indicated by colored markers depending on the type of variant. Translocation calls are shown as colored arcs inside the circos plot. (b) Validated variants associated with the *KMT2A*-r and their interpretation. (c) *KMT2A*::*MLLT10* fusion variant in Access. (d) *KMT2A* and *MLLT10* loci in VIA. (e) Partial putative der(10) based on variants identified by OGM (green rectangle indicates FISH break-apart probe approximate location). (f) Putative KMT2A-MLLT10 fusion protein based on fusion transcript information. MBM: menin-binding motif; LBD: LEDGF-binding domain; SNL1-2: nuclear-localization signals; CxxC: zinc finger-CxxC domain; AF10-like_CC: coiled-coil domain. Numbers in panels c–e correspond to OGM variant calls listed in panel b. *fusion call with no apparent loss of material is an inversion with co-occurence of the insertion of 5′*KMT2A* at the breakpoint in *MLLT10.*

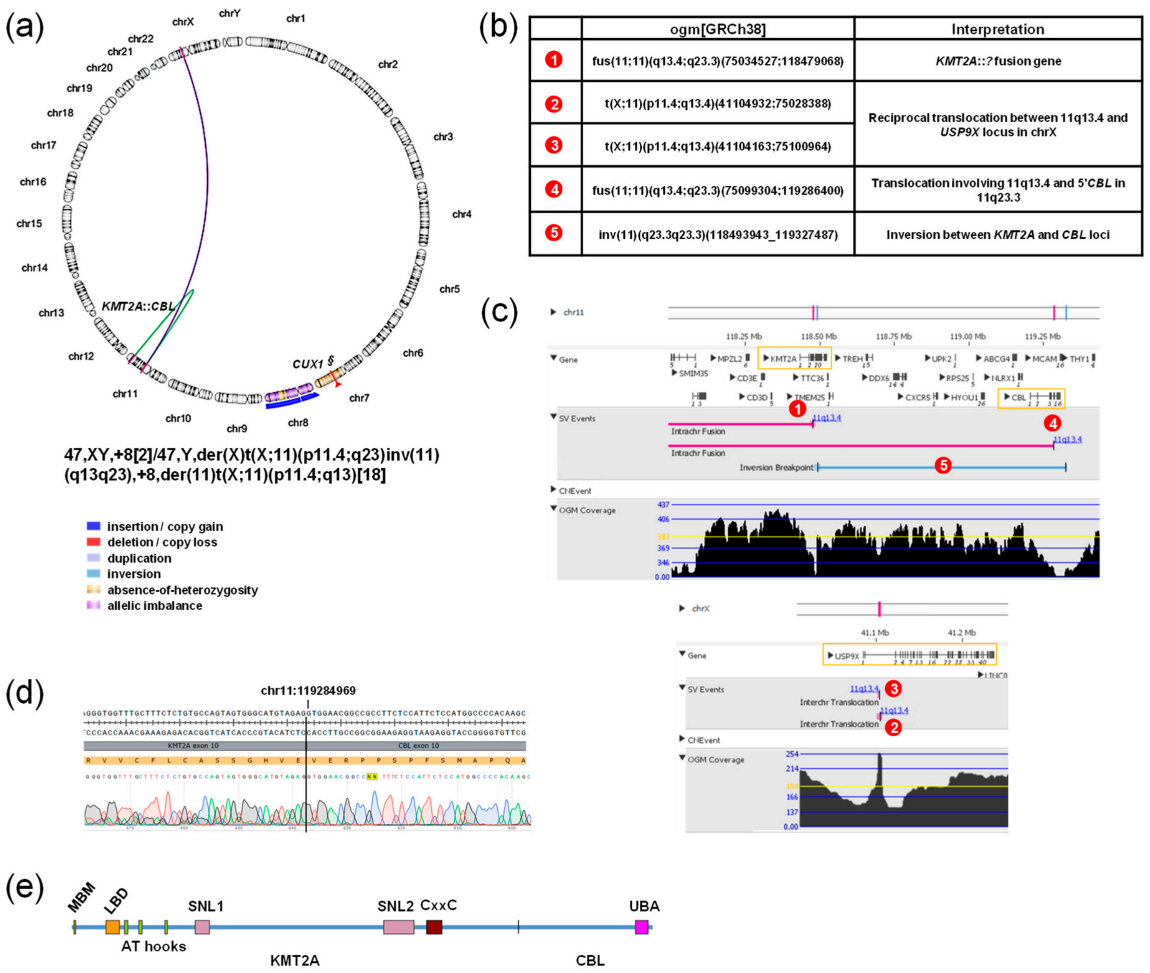

Figure S12. *KMT2A*::*CBL* fusion rearrangement in case 07H114.

(a) Circos plot and karyotype result. Chromosomes are displayed in a circular pattern with the location of variant calls indicated by colored markers depending on the type of variant. Translocation calls are shown as colored arcs inside the circos plot. (b) Validated variants associated with the *KMT2A*-r and their interpretation. (c) *KMT2A* to *CBL* region (11q23.3) and *USP9X* locus (Xp11.4) in VIA. (d) Sanger sequencing confirming expression of a *KMT2A*::*CBL* fusion transcript. (e) Putative KMT2A-CBL fusion protein based on transcript sequencing information. MBM: menin-binding motif; LBD: LEDGF-binding domain; SNL1-2: nuclear-localization signals; CxxC: zinc finger-CxxC domain; UBA: Ubiquitin-associated domain. Numbers in panel c correspond to OGM variant calls listed in panel b. § *CUX1* deletion

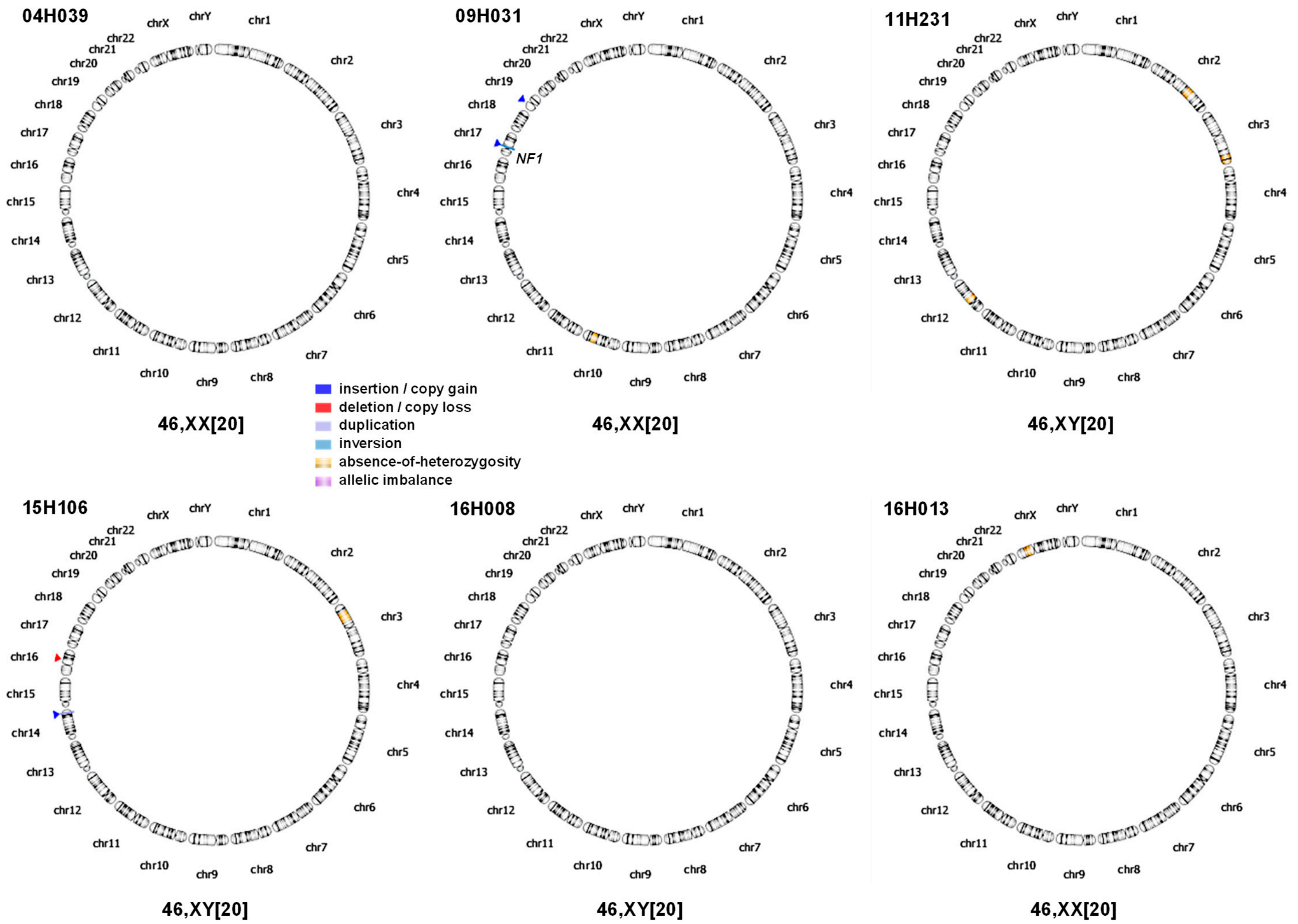

Figure S13. Circos plots of control samples with no *KMT2A* anomaly.
Chromosomes are displayed in a circular pattern with the location of variant calls indicated by colored markers depending on the type of variant. Translocation calls are shown as colored arcs inside the circos plot. Variants determined to be likely benign are not displayed. Validated variants are small regions of absence-of-heterozygosity or large rearrangements (> 500 kb) of unknown significance, except for a rearranged *NF1* allele in case 09H031.

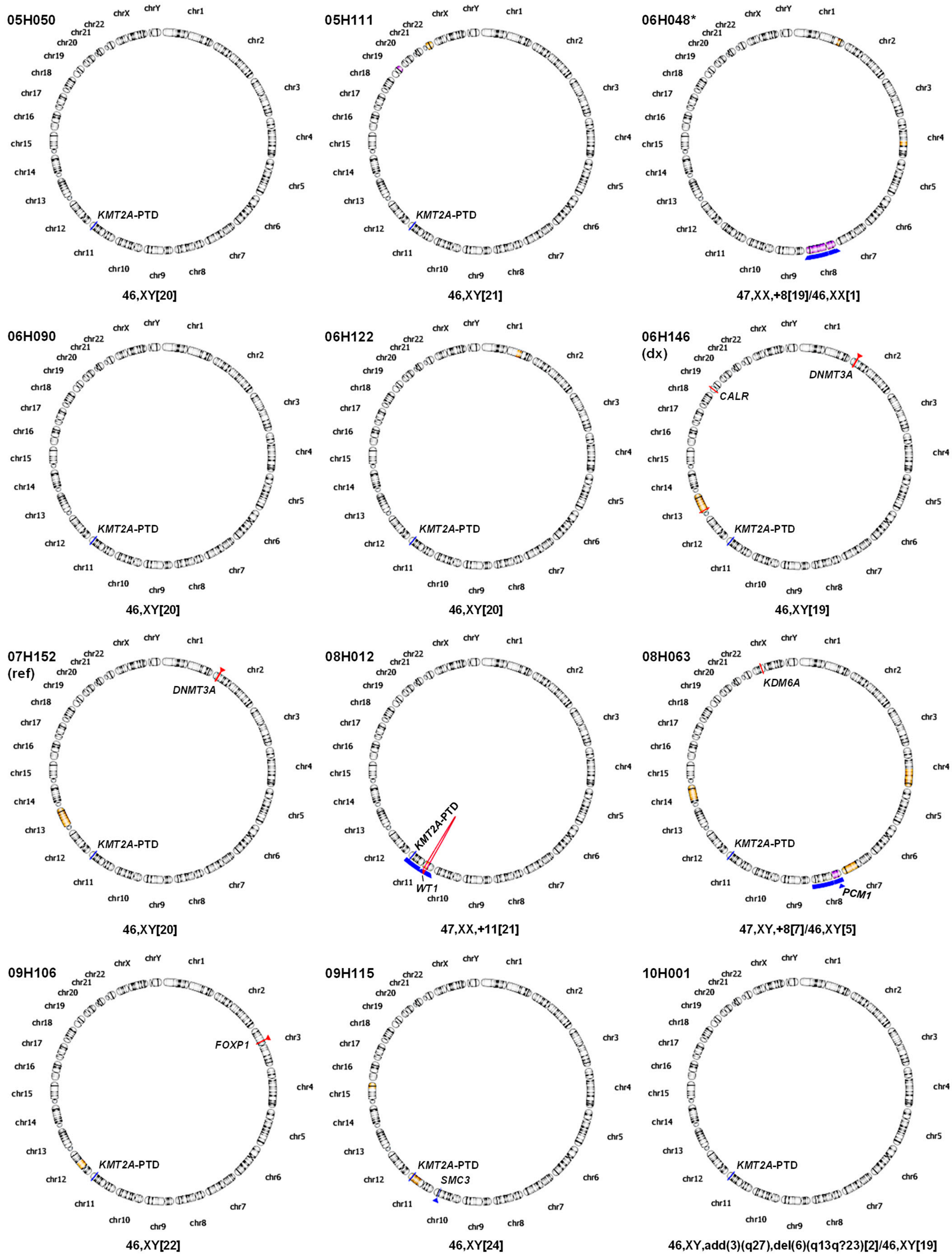

Figure S14. Circos plots of *KMT2A*-PTD cases (continued next page).

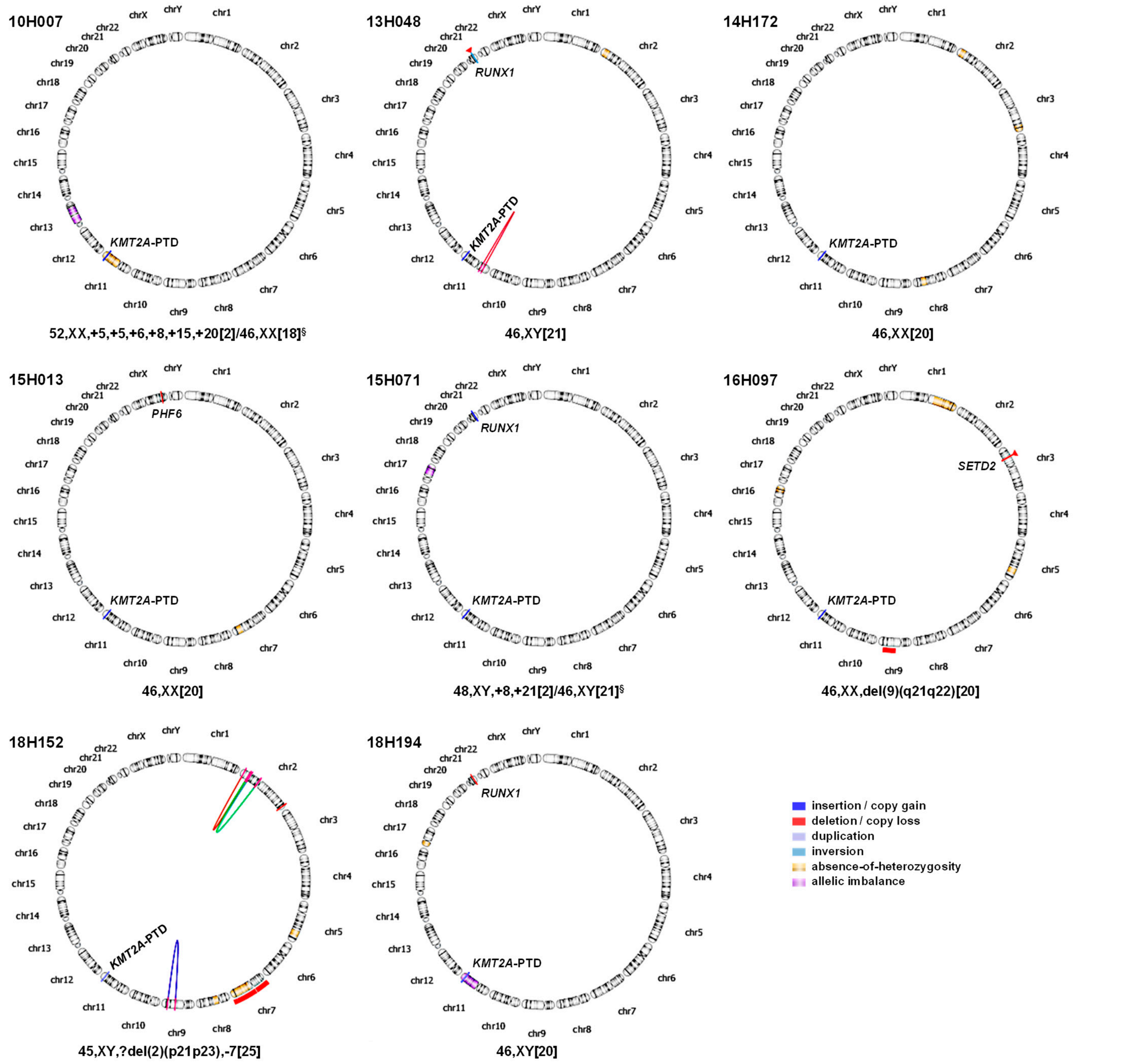

Figure S14. Circos plots of *KMT2A*-PTD cases (continued).

Chromosomes are displayed in a circular pattern with the location of variant calls indicated by colored markers depending on the type of variant. Translocation calls are shown as colored arcs inside the circos plot. Variants determined to be likely benign are not displayed. Variants possibly or likely relevant to leukemic transformation are indicated with a gene name. dx: diagnosis; ref: refractory. *Case 06H048: *KMT2A*-PTD variant was detected only with Guided Assembly – Low Allele Frequency pipeline of analysis. §Cases 10H007 and 15H071: Chromosome gains found in 2 metaphases were not detected by OGM.

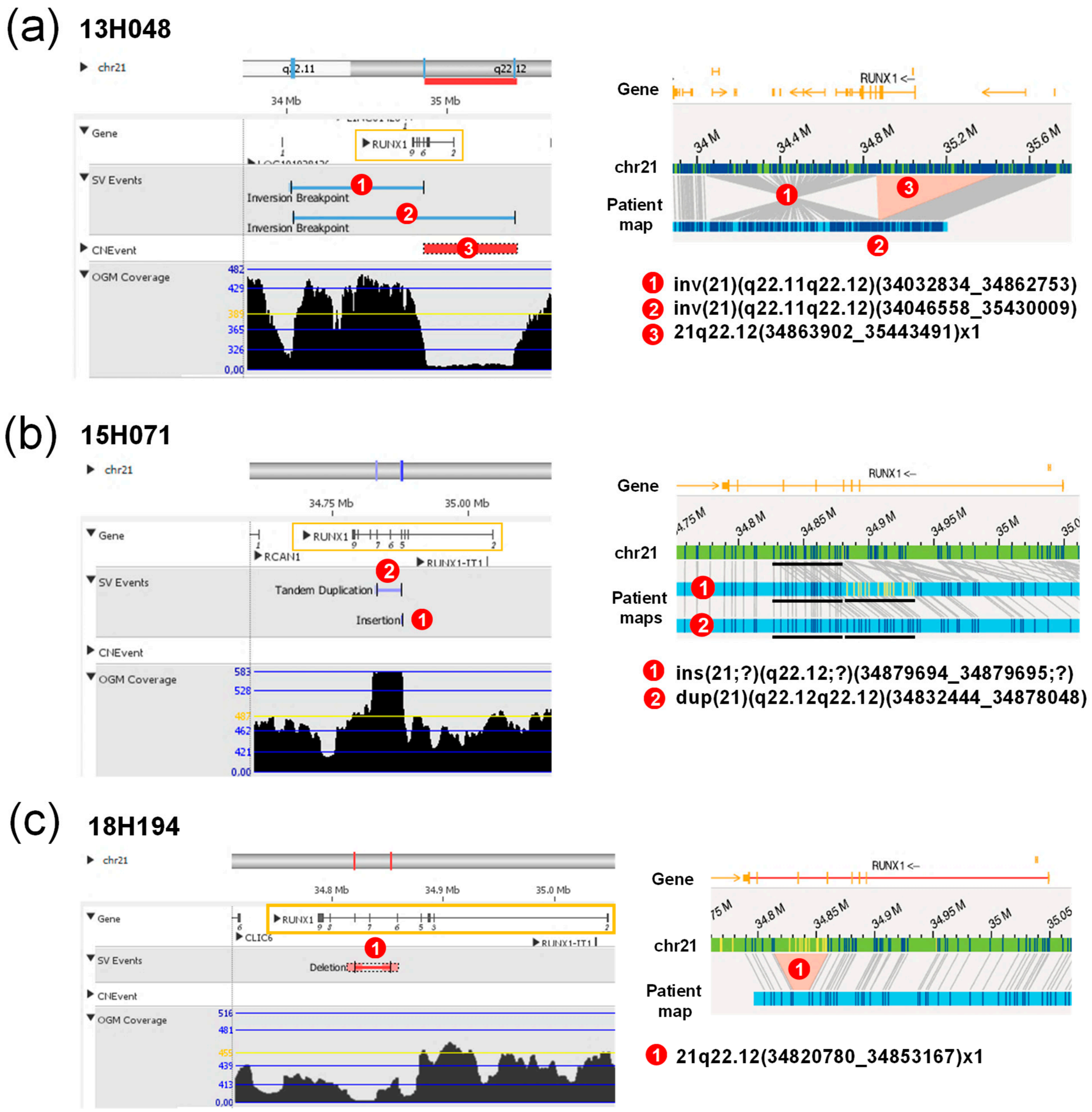

Figure S15. *RUNX1* variants identified in *KMT2A*-PTD cases.
(a) *RUNX1* (NM_001754.5) locus in VIA (left) and Access (right) for case 13H048. The 3 variants identified describe the same rearranged *RUNX1* allele (Guided Assembly analysis results shown). (b) *RUNX1* locus in VIA (left) and Access (right) for case 15H071: a 56.1 kb internal tandem duplication (❶) also detected as an insertion (❷) leading to the duplication of exons 6 and 7. (c) *RUNX1* locus in VIA (left) and Access (right) for case 18H194: a 32.4 kb internal deletion (❶) leading to the loss of exons 7 and 8.

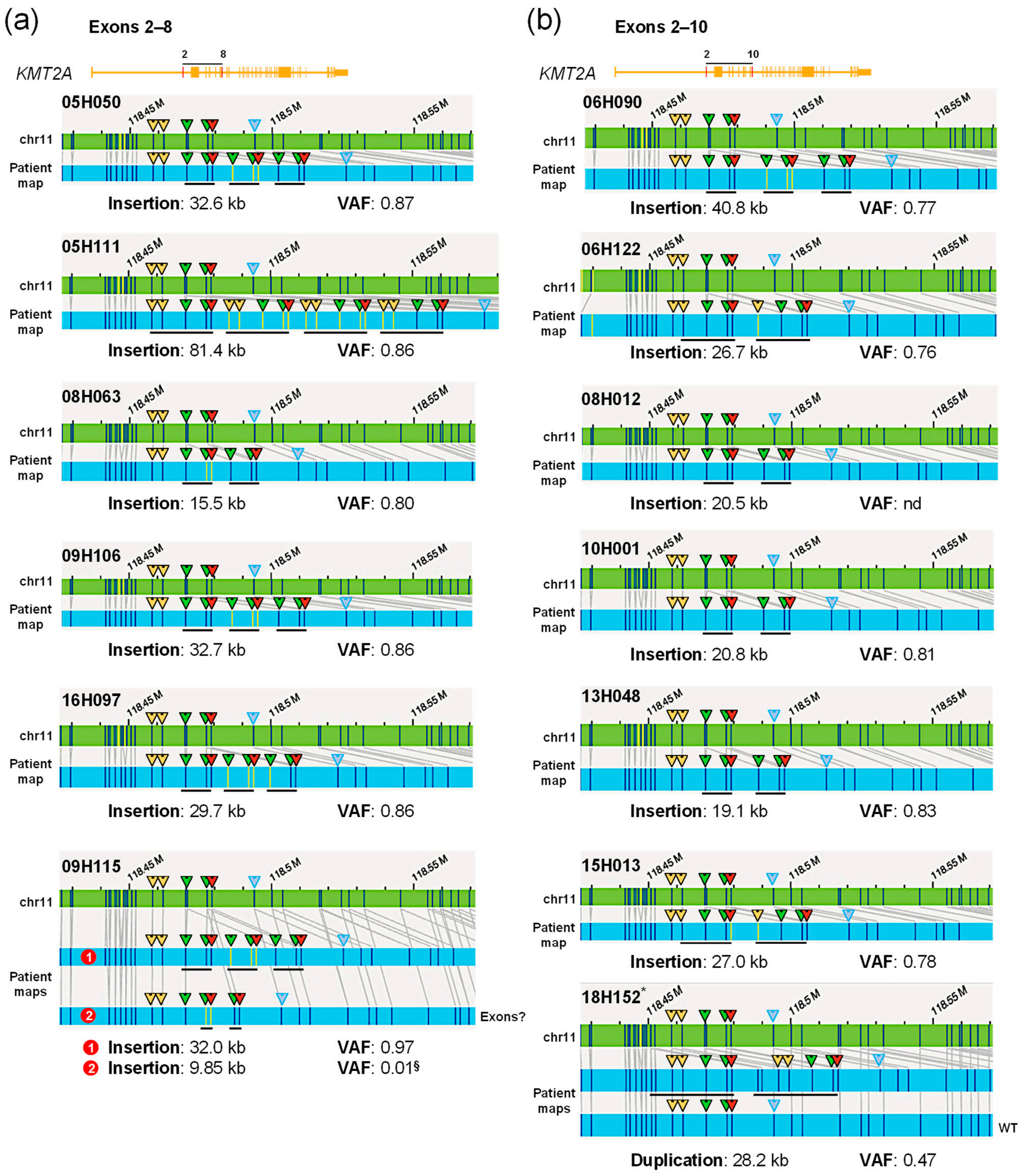

Figure S16. Other *KMT2A*-PTD variants analyzed.
(a) Repeats of exons 2 to 8. (b) Repeats of exons 2 to 10. Colored triangles indicate DLE-1 sites of interest in the characterization of *KMT2A* variants. Variants were identified as insertions or duplications. VAF: Variant Allele Frequency. nd: not determined. *Guided Assembly – Low Allele Frequency pipeline of analysis. § variant not detected with the RNA analysis.
